# Network Pharmacology Analysis and Biological Validation Systemically Identified the Active Ingredients and Molecular Targets of Kudzu Root on Osteoporosis

**DOI:** 10.3390/ijms26031202

**Published:** 2025-01-30

**Authors:** Zhi-Wen Liu, Bo-Bo Zhang, Kevin Wing-Hin Kwok, Xiao-Li Dong, Ka-Hing Wong

**Affiliations:** 1 Research Institute for Future Food, The Hong Kong Polytechnic University, Hong Kong, China; zhiwen.liu@polyu.edu.hk (Z.-W.L.); kwh.kwok@polyu.edu.hk (K.W.-H.K.); 2 Department of Food Science and Nutrition, The Hong Kong Polytechnic University, Hong Kong, China; 3 Guangdong Provincial Key Laboratory of Marine Biology, Department of Biology, College of Science, Shantou University, Shantou 515063, China; bbzhang@stu.edu.cn

**Keywords:** Kudzu root, osteoporosis, network pharmacology, TNF signaling pathway, NF-κB, p38 MAPK

## Abstract

As a traditional medicinal food, Kudzu root (KR) has been proven to be an effective medicine for treating osteoporosis (OP). However, its precise targets and underlying integrated pharmacological mechanisms on OP have not yet been systematically investigated. The aim of the present study was to systemically explore the active ingredients, molecular targets, and ingredient-target network of KR against OP by the methods of network pharmacology followed by biological validation in a glucocorticoid-induced bone loss model of zebrafish. Our results identified a total of 15 active compounds with good pharmacokinetic properties in KR and 119 targets related to OP from correspondent databases, forming an ingredient-target network. Additionally, the protein–protein interaction (PPI) network further identified 39 core targets. Enrichment analyses with functional annotation revealed that the TNF signaling pathway and osteoclast differentiation process were significantly enriched by multi-targets including AKT1, P65, MAPK14, JUN, TNF-α, MMP9, IL6, and IL1B, etc., and served as the critical targets for molecular docking, molecular dynamics simulation, and in vivo experiment validation. These critical targets performed effectively in molecular docking and molecular dynamics, with AKT1, MMP9, and TNF-α exhibiting more prominent binding energy with Coumestrol, Genistein, and Genistein 7-glucoside, respectively. Further experimental validation in a zebrafish model indicated that KR could regulate the expressions of critical targets (AKT1, P65, MAPK14, JUN, TNF-α, and MMP9). This study provides a systemic perspective of the relationships between the active ingredients of KR and their multi-targets in OP, thereby constructing a pharmacological network to clarify the mechanisms by which KR ameliorates OP.

## 1. Introduction

Osteoporosis (OP) is a systemic disease characterized by a decreased bone mineral density and bone strength as well as a damaged bone microstructure, leading to a high frequency of bone fractures and complications. With the aging of the global population, the prevalence of OP in the elderly population is extremely high. The latest study on OP prevalence in China showed that the incidence of OP in individuals aged 40 or older was 5.0% in men and 20.6% in women, while the prevalence of vertebral fracture was 10.5% in men and 9.7% in women. Within the European Union, around 23 million individuals are at high risk of osteoporotic fractures [1,2]. OP is caused by abnormal bone remodeling mediated by bone-forming osteoblasts and bone-resorbing osteoclasts. Clinical drugs for OP include sclerostin inhibitor (Romosozumab), bisphosphonates (Alendronate sodium), receptor activator of nuclear factor κB ligand (RANKL) inhibitors (Denosumab), estrogen (Estradiol valerate), parathyroid hormone (PTH), and related peptide analogues to promote bone formation [3,4]. However, these drugs have certain limitations due to adverse side effects, such as the potential risk of breast cancer, joint and muscle pain, osteonecrosis of the jaw, and some atypical femur fractures [3,5,6]. Moreover, some drugs like PTH must be administered via injection, which leads to many adverse outcomes. Nowadays, medicinal foods, replacing of drugs, are attracting more and more attentions for the treatment of chronic metabolism diseases like OP.

Traditional medicinal foods have been used in China for thousands of years and many of them are widely used in the treatment of OP [7,8,9]. Kudzu root (KR), a dried radix of *Pueraria lobata* (Willd.) Ohwi, named “Gegen” is indigenous to Southeast Asia and has been used as a food source, animal fodder, and medicinal remedy for thousands of years. Its traditional medicinal efficacy includes the prevention and treatment of endocrine system and cardiovascular diseases, such as diabetes, hypertension, and cerebral ischemia and reperfusion [10,11]. With further intensive research, the anti-osteoporotic efficacy of KR has increasingly attracted attention due to the estrogenic-like effects of its bioactive compounds. KR extract can ameliorate postmenopausal bone loss by promoting estrogenic activity and decreasing bone turnover markers in ovariectomized rats [12]. Another study has found that KR can completely prevent bone loss in ovariectomy (OVX) mice without inducing estrogenic effects in the uterus. These effects appear to be associated with the high content of the isoflavones daidzein and genistein [13]. Meanwhile, puerarin, the main isoflavone glycoside in KR, can ameliorate OVX-induced mice by suppressing osteoclastogenesis via inhibition of the TNF receptor-associated factor 6 (TRAF6)/ROS-dependent mitogen-activated protein kinase (MAPK)/NF-κB signaling pathways and concurrently promotes the proliferation and differentiation of osteoblasts by stimulating osteoprotegerin and inhibiting RANKL and interleukin 6 (IL6) production [14,15,16]. Despite these discoveries, the precise targets and underlying integrated pharmacological mechanisms by which KR prevents OP have not yet been systematically investigated.

The medicinal/functional foods such as KR exist as a complex system with numerous targets and synergistic or antagonistic interactions between their components and possess “multi-component, multi-target, and multi-pathway” characteristics. With the rapid development of computer technology, bioinformatics, and systems biology theory, network pharmacology has so far evolved as a powerful research strategy to explore complex mechanisms of Traditional Chinese Medicine (TCM) or medicinal foods [17,18,19]. The “network target, multi-components” key concept of network pharmacology can methodically clarify the molecular mechanism of the action of medicinal/functional foods on different diseases and facilitate the development of evidence-based medicine and the discovery of novel medicinal/functional foods [20,21,22,23].

**Figure 1:**
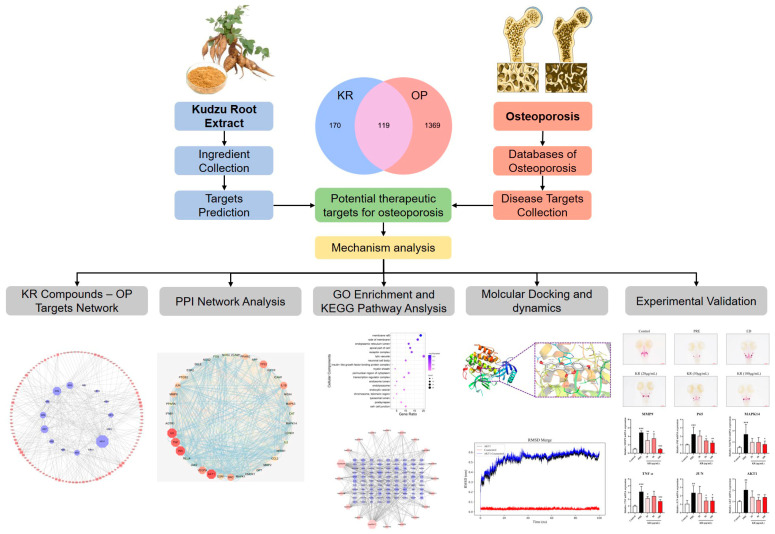
Flowchart showing the network pharmacological and experimental studies for the investigation of the anti-osteoporotic effects of KR. ### *p* < 0.001 compared with control. * *p* < 0.05; ** *p* < 0.01; *** *p* < 0.001 compared with PRE.

The aim of this research was to identify the targets of KR in treating OP using network pharmacology to investigate the network relationship between the drug, its targets, and associated signaling pathways. Several core targets were experimentally validated, offering a scientific foundation for elucidating the mechanism of KR against OP and for the development of medicinal/functional foods (Figure 1).

## 2. Results and Discussion

### 2.1. Screening of Active Compounds in KR and Construction of Compound-Target Network

**Figure 2:**
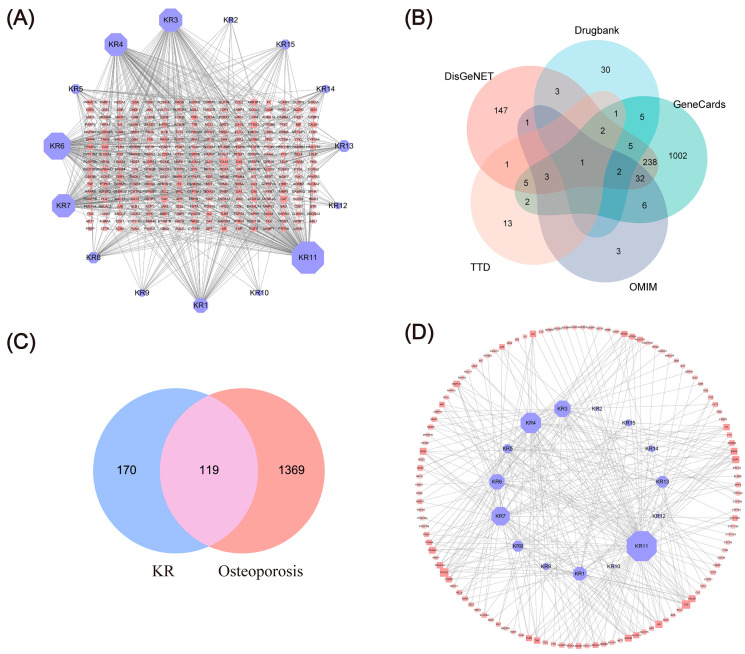
Acquisition of KR-OP-targeted proteins. (**A**) Network of targets predicted using the KR-derived compounds. (**B**) The Venn diagram of OP-related targets. (**C**) The Venn diagram of 119 anti-OP targets of KR. (**D**) The compound-target network implicated in the OP treatment using KR. Purple nodes indicate active compounds, and red nodes indicate target proteins.

It is generally accepted that the core compounds in medicinal/functional foods make great contributions to the therapy of diseases. In this study, 34 KR compounds were obtained from the public databases, and among them, 15 KR bioactive compounds were screened through the pharmacokinetic properties of ADME (Absorption, Distribution, Metabolism, and Excretion) (Table 1). We predicted 281 targets for these 15 active compounds based on pharmacophore and drug–drug similarity, and then the 15 active compounds-281 protein targets network (Table S1) was constructed (Figure 2A). Network analysis showed that Genistein (KR11, degree = 124), Dalbergenone (KR6, degree = 101), and Formononetin (KR7, degree = 84) had the most connections to various targets.

The integration of data from the multiple public databases retrieved a total of 1488 targets related to OP (Figure 2B). Comparing these OP targets to the predicted KR targets, 119 common targets were filtered as predicted targets of 15 KR active compounds against OP (Figure 2C). Furthermore, a network comprising 298 edges and 134 nodes was generated using the 15 active compounds targeting the OP-related protein targets (Figure 2D and Table S2). Genistein (KR11, degree = 65), Daidzein (KR4, degree = 41), Formononetin (KR7, degree = 36), Coumestrol (KR3, degree = 31), and Dalbergenone (KR6, degree = 28) were recognized as possessing the maximum number of connections to various targets, suggesting that these compounds are most likely the crucial ingredients in KR. Additionally, some proteins were targeted by multiple compounds, Carbonic Anhydrase II (CA2, degree = 11) and Prostaglandin G/H synthase 2 (PTGS2, degree = 11) were modulated by 11 compounds, including Puerarin (KR13), Coumestrol (KR3), Daidzein (KR4), Daidzin (KR5), and Formononetin (KR7), etc.

The classification of compounds and KR compound-target network analysis indicated that the flavonoid compounds, such as Coumestrol, Daidzein, Daidzin, Formononetin, Genistein, and Puerarin, are the primary active components in KR for the treatment of OP, and these active components in the component-target network have high degree values. In addition, the same target can be linked to multiple active ingredients. For example, TNF-α can be linked to Daidzein, Daidzin, Formononetin-7-glucoside, Genistein 7-glucoside, Genistein, and Puerarin. These results were in line with previous findings that Puerarin and Formononetin could inhibit osteoclast differentiation through suppressing the expression of the activating transcription factor AP-1 and osteoclast-specific genes [Nuclear factor of activated T-cell cytoplasmic 1 (NFATc1), Matrix metalloproteinase 9 (MMP9), Cathepsin K (CTSK), c-Fos, and Tartrate-resistant acid phosphatase (TRAP)] [14,24]. Moreover, Daidzein could stimulate osteogenesis, by facilitating proliferation, differentiation, and anti-apoptosis in human osteoblast-like MG-63 cells. Coumestrol could also promote proliferation and osteoblastic differentiation in rat bone marrow stromal cells [25,26]. Therefore, all these findings indicated that Coumestrol, Daidzein, Puerarin, and Formononetin may be the main bioactive ingredients responsible for the efficacy of KR in preventing OP.

### 2.2. Construction of the PPI Network for the Anti-OP Targets of KR

**Figure 3:**
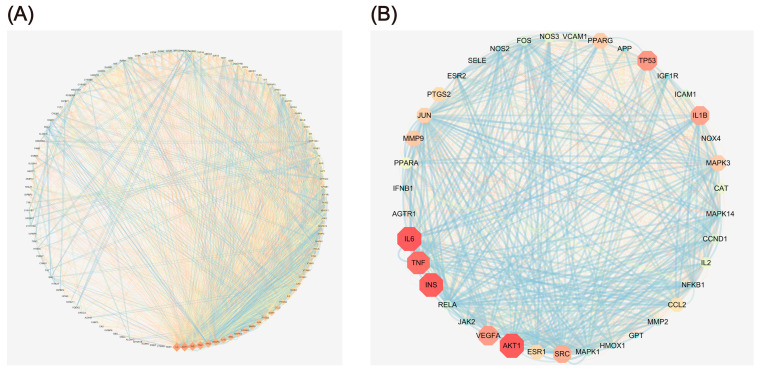
Reconstructed PPI network. (**A**) The PPI network constructed using Cytoscape. (**B**) The cluster generated from (**A**) based on MCODE analysis; different colors signify the degree. Node size is proportional to the degree of interaction.

The identification of OP-related targets for the core components of KR was achieved using a protein–protein interaction (PPI) network, which is extensively used to illustrate the complex interactions that occur among different proteins in complicated diseases. In this study, the gene names of 119 anti-OP targets for KR were entered into the STRING database for constructing a PPI network by using Cytoscape-3.8.0. As shown in Figure 3A and Table S3, the constructed PPI network included 2848 edges and 118 nodes. The core targets of the PPI network were further screened by using a Molecular Complex Detection (MCODE) algorithm [27] to produce a highly interconnected sub-network. As shown in Figure 3B and Tables S4 and S5, the sub-network composed of 1190 edges and 39 nodes; the average betweenness centrality, average degree centrality, and average closeness centrality of the cluster were 0.0200, 47.0256, and 0.6236, respectively. The degree centrality, betweenness centrality, and closeness centrality values of the following 13 targets were higher than the averages: AKT1, IL6, INS, TNF-α, TP53, VEGFA, IL1B, SRC, MAPK3, MMP9, PPARG, JUN, and PTGS2, which were identified as core anti-OP targets of KR. Interestingly, the top targets of KR for OP, including AKT1, TNF-α, IL6, and IL1B, were closely related to the process of inflammation, among which, TNF-α is a critical upstream regulator. Previous studies have shown that RANKL expression can be induced by TNF-α and IL-6, which has been proven to impair bone remodeling by activating osteoclasts [28].

### 2.3. Gene Ontology Enrichment Analysis of the Anti-OP Targets of KR

**Figure 4:**
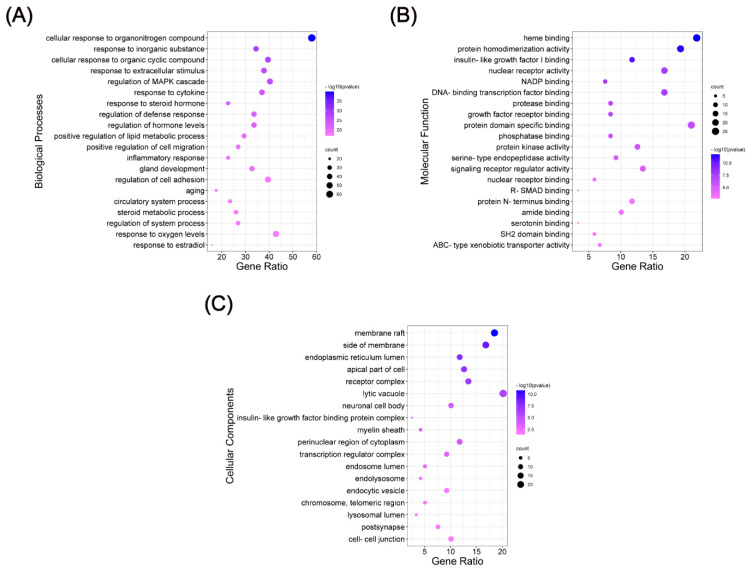
GO enrichment analysis of the anti-OP targets of KR. (**A**) Biological processes, (**B**) molecular function, and (**C**) cellular components.

To expound the corresponding pathological events involved in the biological processes of these major targets during the progression of OP, gene ontology (GO) enrichment analyses [biological processes (BP), molecular function (MF), and cellular component (CC)] of the 119 predicted targets were performed. As shown in Figure 4, the top 20 enriched BP, MF, and CC terms were identified. Comprehensive details about the GO analysis are provided in Tables S6–S8. The results of BP analysis indicated that the process of KR preventing OP is closely related with the cellular response to organonitrogen compound, response to inorganic substance, cellular response to organic cyclic compound, response to extracellular stimulus, and regulation of MAPK cascade. Studies showed that MAPK signaling pathway triggered by M-CSF mainly regulates the proliferation of osteoclast precursor, while RANKL-induced activation of MAPK signaling pathway is chiefly implicated in osteoclast differentiation [29,30]. MF analysis revealed that related targets mainly centered on heme binding, protein homodimerization activity, and insulin-like growth factor I binding, etc. Moreover, CC analysis revealed that the anti-OP targets of KR mainly focused on membrane raft, side of membrane, and endoplasmic reticulum lumen, etc. However, these predicted BP, MF, and CC in the top terms poorly reflect the direct effect of KR for the treatment of OP. Therefore, Kyoto Encyclopedia of Genes and Genomes (KEGG) pathway enrichment analysis was conducted to further elucidate the mechanism of action of KR in the prevention of OP.

### 2.4. Pathway Enrichment Analysis and Compound-Target-Pathway Network Construction

**Figure 5:**
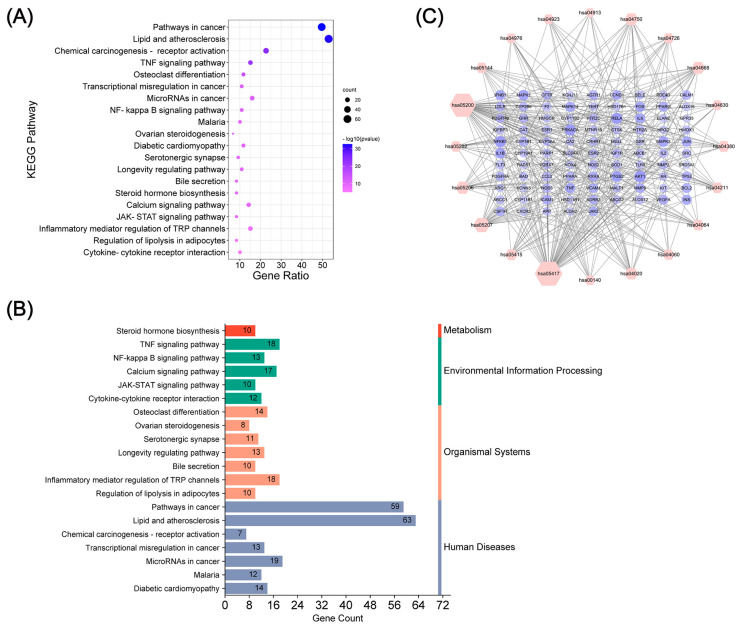
KEGG pathway enrichment analysis of the anti-OP targets of KR. (**A**) Top 20 KEGG pathways. (**B**) The distribution of pathways for the anti-OP targets of KR annotated in the KEGG database. (**C**) The target-pathway network implicated in the mechanism of KR in OP treatment. Red nodes indicate the pathways, purple nodes indicate the targets involved in these pathways. The interactions between the targets and the pathways are represented by edges, and the node is proportional to the degree of interaction.

**Figure 6:**
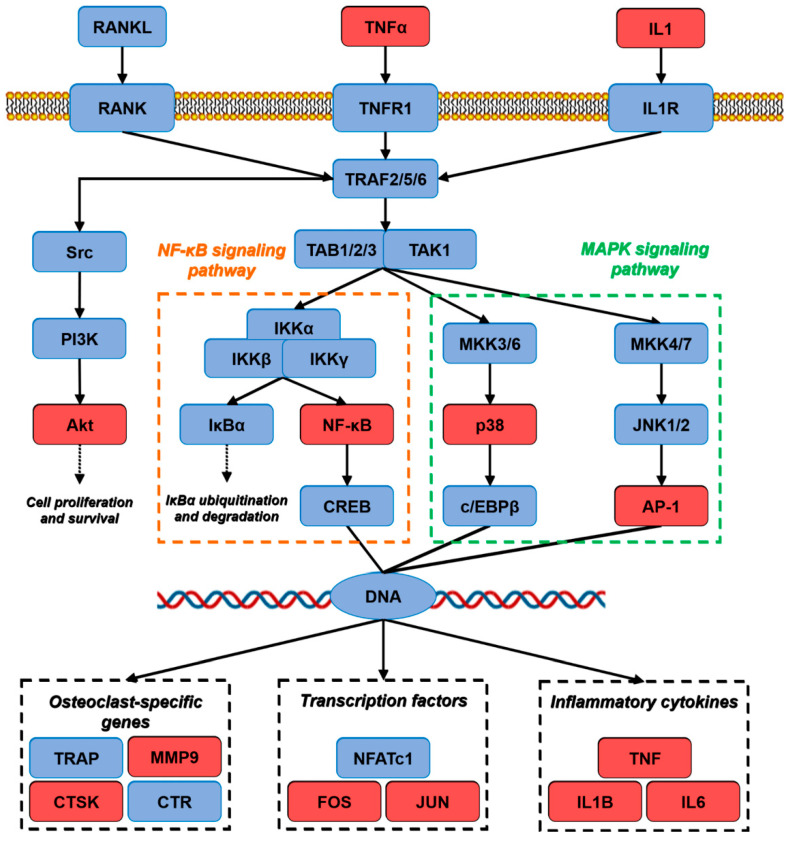
Distribution of the target proteins of KR on the predicted pathway. The red nodes are putative target proteins of KR, while the blue nodes are relevant targets in the pathway.

To investigate the representative signaling pathways associated with the anti-OP targets of KR, we conducted a KEGG pathway enrichment analysis for the 119 KR targets at a significance threshold of *p* < 0.01 (Figure 5A). The annotation and distribution of the enrichment pathway are shown in Figure 5B and Table 2. A target–pathway network which included 371 edges and 115 nodes was established according to the number of targets involved in various pathways (Figure 5C, Table S9). We found that the majority of targets were mostly involved in pathways in lipid and atherosclerosis, pathways in cancer, and chemical carcinogenesis - receptor activation. Additionally, the targets involved in the greatest number of signaling pathways were AKT1, RELA (P65), and PRKACA, which in turn were involved in the 11th, 10th, and 10th pathways, respectively (Figure 5C). The findings from the target-pathway network and KEGG enrichment analysis indicated that the TNF signaling pathway and osteoclast differentiation pathway in the top five enriched pathways were closely related to pathological process of OP (Figure 6). Based on the target-pathway network data, we found that the effective components of KR mainly act on AKT1, RELA (P65), NFKB1, TNF-α, JUN, MAPK14 (P38), IL6, IL1B, MMP9, MAPK1, and MAPK3, which are mainly mapped to key pathways including the TNF signaling pathway and osteoclast differentiation pathway.

It is well known that RANKL, an upstream cytokine for osteoclastogenesis, is needed for osteoclast differentiation. RANKL promotes downstream pathways such as NF-κB, MAPK14 (p38), and JNK to enhance osteoclastogenesis in osteoclast precursors via its receptor c-fms and RANK [31]. Activation of NF-κB and MAPKs pathways induces NFATc1 and AP-1, crucial transcriptional regulators of osteoclast development [32]. The AP-1 complex composed of Fos and JUN is a transcriptional partner of NFAT in osteoclastogenesis, which induces osteoclast-specific genes such as TRAP, CTSK, MMP9, and CA2 [33,34]. Numerous studies have demonstrated that the TNF signaling pathway plays a crucial role in the pathogenic processes of OP. Increased TNF level could synergistically promote RANKL-induced osteoclasts formation through activation of NF-κB signaling pathway [35]. Importantly, other studies found that the PI3K/Akt pathway associated with multiple cellular processes is the upstream signaling of the NF-κB pathway, and inhibition of PI3K/Akt totally blocked the synergistic effects of TNF-α on NF-κB activation as well as osteoclast formation [36,37]. Therefore, TNF-α as an upstream mediator of osteoclast differentiation could promote RANKL-induced osteoclastogenesis through activation of NF-κB, which was mediated by PI3K/Akt signaling. Interestingly, these previous findings were consistent with our present results from PPI analysis that AKT1, TNF-α, and IL6, etc., played an important role in the cluster interacting with other KR targets. Based on the KEGG pathway enrichment analysis, we can clearly confirm that the mechanism of KR in the treatment of OP is mainly via the TNF-α/AKT/NF-κB/p38 MAPK signaling axis.

### 2.5. Molecular Docking Analysis for the Interaction Between KR Active Components and Predicted Anti-OP Targets

**Figure 7:**
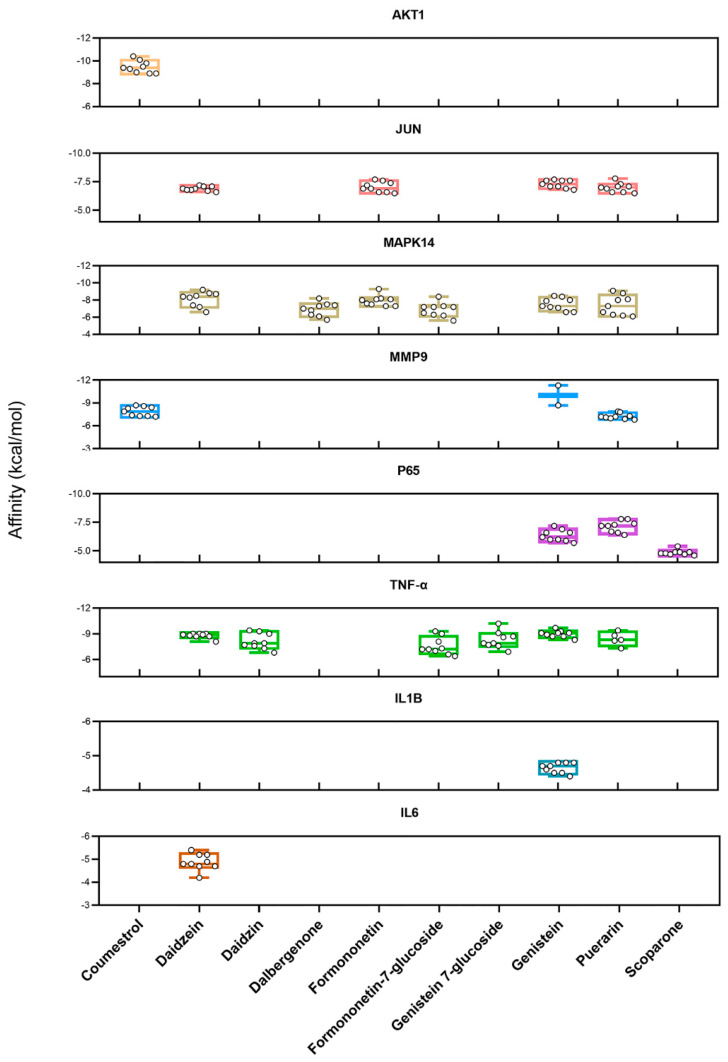
Docking models of enriched targets of the TNF and osteoclast differentiation pathway with their corresponding active compounds are shown as boxplots. Each point represents the affinity of the docking pose between a compound (ligand) and an enriched target (receptor).

**Figure 8:**
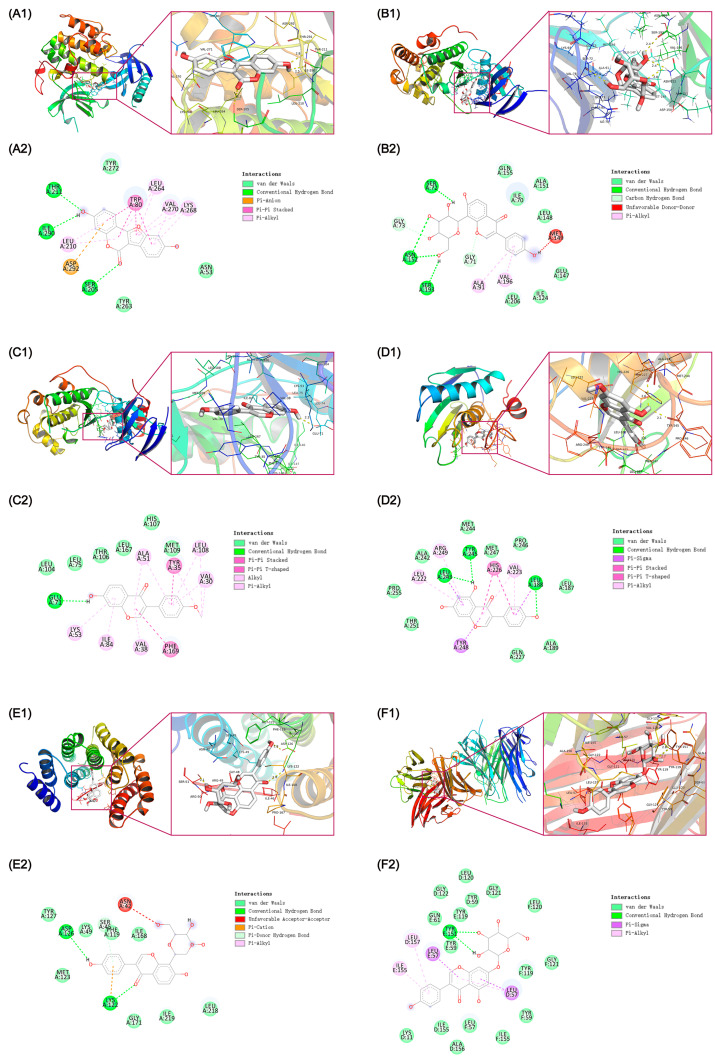
Molecular docking diagram for complexes of the KR active compounds-protein targets. Binding mode of Coumestrol (KR3) to AKT1 (**A1**), Puerarin (KR13) to JUN (**B1**), Formononetin (KR7) to MAPK14 (**C1**), Genistein (KR11) to MMP9 (**D1**), Puerarin (KR13) to P65 (**E1**), and Genistein 7-glucoside (KR9) to TNF-α (**F1**). (**A2**–**F2**): Two dimensional patterns of bond, respectively.

Based on the results of PPI analysis and bioinformatics enrichment analysis, the molecular docking was further performed to examine the interactions between the active compounds and their potential targets. The interactions of the eight crucial targets (AKT1, P65, MAPK14, JUN, TNF-α, MMP9, IL6, and IL1B) with the active components of KR were analyzed using AutoDockTools-1.5.6 and Autodock Vina-1.1.2. The free binding energy of protein targets with their active compounds was detailed in Table 3 and Figure 7. A docking score of 0 kcal/mol or lower generally indicates spontaneous interaction between the ligand and the receptor, a score of −4.0 kcal/mol or lower suggests good docking affinity, and a score of −7.0 kcal/mol or lower suggests strong docking affinity [38,39,40]. In this study, we found that AKT1, P65, MAPK14, JUN, TNF-α, and MMP9 exhibited significantly higher affinity with active compounds. The spatial coordinates of the compound–target complexes with the strongest free binding affinity and their binding modes were visually illustrated using PyMOL-1.7.2.1 and Discovery Studio-2020 (Figure 8).

The free binding energy of the Coumestrol (KR3)-AKT1 complex was −10.4 kcal/mol. As shown in Figure 8(A1,A2), Coumestrol exhibited stable binding to AKT1 through hydrogen bonds with THR-211, SER-205, and ILE-290 residues. Coumestrol also interacted with TYR-272, TYR-263, and ASN-53 residues via van der Waals forces. Meanwhile, Coumestrol formed Pi–Pi stacked, Pi-Anion, and Pi-Alkyl bonds with TRP-80, ASP-292, LEU-264, LEU-210, VAL-270, and LYS-268 residues of AKT1. Compared with other TCM-derived active compounds with anti-OP activity (e.g., Quercetin, Luteolin, and Kaempferol), Coumestrol exhibited a stronger binding affinity with AKT1 [41].

The free binding energy of Puerarin (KR13) with JUN was −7.8 kcal/mol. As shown in Figure 8(B1,B2), Puerarin bound to JUN through hydrogen bonds with SER-72, ASN-152, and SER-193 residues, the carbon hydrogen bonds with GLY-73 and GLY-71 residues, and the unfavorable donor–donor interaction with MET-149 residue. Simultaneously, Puerarin formed Pi–Alkyl bonds with ALA-91 and VAL-196 residues, and interacted with GLN-155, ILE-70, ALA-151, LEU-148, GLU-147, ILE-124, and LEU-206 residues via van der Waals forces.

The free binding energy of Formononetin (KR7) with MAPK14 was −9.3 kcal/mol. As shown in Figure 8(C1,C2), Formononetin bound to MAPK14 with GLU-71 residue through a hydrogen bond, LYS-53, ILE-84, VAL-38, ALA-51, LEU-108, and VAL-30 residues through Pi-Alkyl and Alkyl bonds, and formed Pi–Pi stacked and Pi–Pi T-shaped bonds with TYR-35 and PHE-169 residues. Formononetin also interacted with LEU-104, LEU-75, THR-106, LEU-167, HIS-107, and MET-109 residues via van der Waals forces.

The free binding energy of the Genistein (KR11)-MMP9 complex was −11.3 kcal/mol. As shown in Figure 8(D1,D2), Genistein formed hydrogen bonds with MMP9 residues LEU-243, TYR-245, and LEU-188, the Pi–Sigma, Pi-Pi stacked, Pi-Pi T-shaped, and Pi-Alkyl bonds through hydrophobic interactions with TYR-248, LEU-188, and LEU-243, HIS-226, LEU-222, ARG-249, and VAL-223 residues. Meanwhile, Genistein interacted with THR-251, PRO-255, ALA-242, MET-247, MET-244, PRO-246, LEU-187, ALA-189, and GLN-227 residues through van der Waals forces.

The free binding energy of the Puerarin (KR13)-P65 complex was −7.8 kcal/mol. As shown in Figure 8(E1,E2), Puerarin bound to P65 with LYS-122 and ASP-126 residues through hydrogen bonds, ASN-42 residue through unfavorable acceptor–acceptor interaction, and formed Pi-Cation and Pi-Donor hydrogen bonds with LYS-122 and SER-45 residues. Puerarin also interacted with MET-123, TYR-127, LYS-49, PHE-119, LEU-218, ILE-219, ILE-168, and GLY-171 residues via van der Waals forces.

The free binding energy of Genistein 7-glucoside (KR9) with TNF-α was −10.2 kcal/mol. As shown in Figure 8(F1,F2), Genistein 7-glucoside formed a hydrogen bond with TYR-151 residue and Pi–Sigma and Pi-Alkyl bonds with LEU-57, LEU-157, and ILE-155 residues of TNF-α. Genistein 7-glucoside also interacted with LYS-11, ILE-155, ALA-156, LEU-57, TYR-59, TYR-119, GLY-121, LEU-120, GLY-122, and GLN-61 residues through van der Waals forces.

In this study, we found that Formononetin and Genistein demonstrated a greater binding affinity for MAPK14 and MMP9, respectively, compared to Quercetin and Luteolin [42,43]. Additionally, Genistein 7-glucoside exhibited a stronger binding affinity with TNF-α compared to the multiple active compounds of Radix Paeoniae Alba which is a TCM alleviating inflammation [44]. Overall, the free binding affinity of the six complexes were <−7 kcal/mol, specifically, the binding energy of Coumestrol, Formononetin, Genistein, and Genistein 7-glucoside with their corresponding targets were <−9 kcal/mol. These results suggested that KR active compounds are closely bound to the predicted targets, which confirmed the accuracy of target prediction.

The well-founded molecular docking results indicated that five compounds (Coumestrol, Formononetin, Genistein 7-glucoside, Genistein, and Puerarin) exhibited a strong binding affinity with their corresponding targets. Other active compounds targeting AKT1, P65, MAPK14, JUN, TNF-α, and MMP9 were also predicted, and the results showed that the MAPK14, TNF-α, and JUN were targeted by the majority of the bioactive components of KR, with P65, MMP9, and AKT1 following, further indicating that the bioactive components of KR synergistically exert an inhibitory effect on osteoclast differentiation. In the meantime, it is worth noting that flavonoid and isoflavone, the main active ingredients of KR, are the major group of phytoestrogens related to physiological effects. Therefore, the incidence of estrogenic-like side effects in patients undergoing KR administration should be considered in future clinical trials.

### 2.6. Molecular Dynamics Analysis for the Stability of KR Active Components-OP Targets Complexes

**Figure 9:**
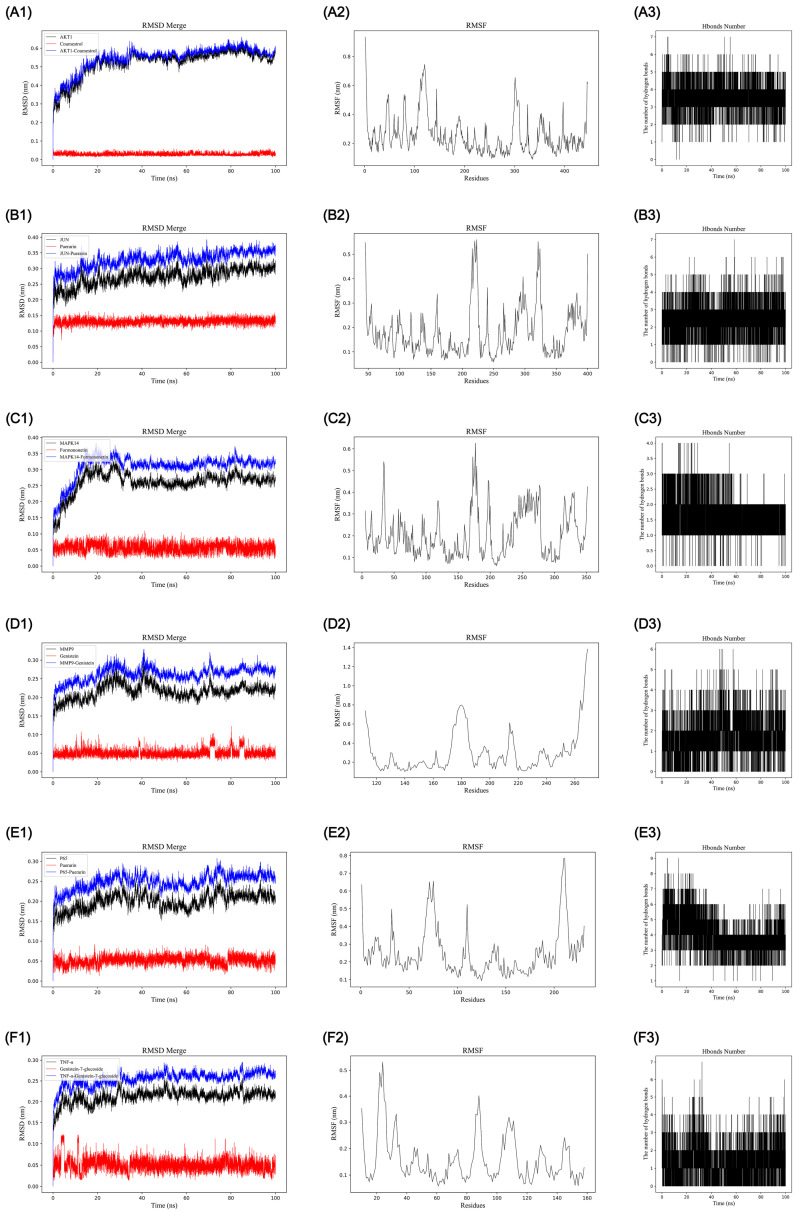
Molecular dynamics simulation of the Coumestrol-AKT1 (**A1**–**A3**), Puerarin-JUN (**B1**–**B3**), Formononetin-MAPK14 (**C1**–**C3**), Genistein-MMP9 (**D1**–**D3**), Puerarin-P65 (**E1**–**E3**), and Genistein 7-glucoside-TNF-α (**F1**–**F3**) complexes. (**A1**–**F1**) RMSD curve. (**A2**–**F2**) RMSF curve. (**A3**–**F3**) Hydrogen bonds.

To further evaluate the influence of KR active components on OP targets, molecular dynamics simulations were conducted to determine the stability of the Coumestrol-AKT1 (Complex 1), Puerarin-JUN (Complex 2), Formononetin-MAPK14 (Complex 3), Genistein-MMP9 (Complex 4), Puerarin-P65 (Complex 5), and Genistein 7-glucoside-TNF-α (Complex 6) complexes obtained from molecular docking in 100 ns. The Root Mean Square Deviation (RMSD) is a statistic used to evaluate the stability of a simulated system [45]. As shown in Figure 9(A1–F1), the complexes 1, 2, 3, 4, 5, and 6 reached equilibrium after 35 ns, 20 ns, 30 ns, 40 ns, 30 ns, and 5 ns, respectively. Meanwhile, the RMSD averages after stabilization of the complexes 1–6 were 0.58 nm, 0.34 nm, 0.32 nm, 0.27 nm, 0.25 nm, and 0.26 nm, respectively. These results indicated that Coumestrol, Formononetin, Genistein, and Genistein 7-glucoside could generate a stable complex system with AKT1, MAPK14, MMP9, and TNF-α, respectively, while Puerarin could also form a stable complex with JUN and P65, which was consistent with the molecular docking results. The Root Mean Square Fluctuation (RMSF) is a metric for assessing the dynamics of proteins, which reflects the movement amplitude of the amino acid residues [46]. A high RMSF suggested that the structure regions of the amino acid residue were generally more flexible during the molecular dynamic simulation process. As shown in Figure 9(A2), residues 1–10, 100–130, 295–310, and 435–446 of Coumestrol-AKT1 (Complex 1) exhibited greater flexibility, which may be potential regions adjacent to the active site involved in substrate binding. Similarly, the results showed that complexes 2–6 also have multiple residue regions with high flexibility (Figure 9(B2–F2)). This suggests that these flexible residue regions may facilitate the binding processes between KR active compounds and OP protein targets, contributing to functional adaptability. Conversely, lower RMSF values in other areas indicated structural rigidity within the target protein, that preserves the integrity of the receptor structure in the complex. The hydrogen bond that forms between the receptor and the ligand contributes to the stability of the complex. As shown in Figure 9(A3–F3), the results revealed that the hydrogen bond numbers for the complexes 1–6 were 0–7, 0–7, 0–4, 0–6, 0–9, and 0–7, respectively, indicating the existence of stable hydrogen bonds in the complexes 1–6.

The Gibbs free energy graphic was utilized to depict the stability of the receptor–ligand complexes [47]. Figure S1 shows the 3D (A1–F1) and 2D (A2–F2) Gibbs energy landscape of the complexes 1–6. The blue regions signify a low-energy conformational state, indicating stable complex system. In this study, we found that complexes 1, 2, and 6 obviously exhibited a single and distinct minimum energy cluster in both 3D and 2D morphologies. Furthermore, the free energy stability of the complexes 1–6 was calculated using MM/PBSA method [48]. As shown in Table 4, the complexes 1, 2, 3, 4, 5, and 6 had a total binding free energy of −27.82 ± 1.82, −29.57 ± 3.34, −26.96 ± 3.14, −28.76 ± 1.51, −30.85 ± 3.85, and −40.46 ± 3.41 kJ/mol, respectively. These low-energy states indicated the formation of stable binding between the active compounds and the protein targets.

In summary, the results of molecular dynamics simulations indicated that Coumestrol, Formononetin, Genistein, Puerarin, and Genistein 7-glucoside could stably bind to AKT1, MAPK14, MMP9, and TNF-α, respectively, while Puerarin showed stable binding to JUN and P65. These findings were aligned with the results of molecular docking and network pharmacology.

### 2.7. Targets Validation for the Anti-OP Effect of KR in Zebrafish Model

**Figure 10:**
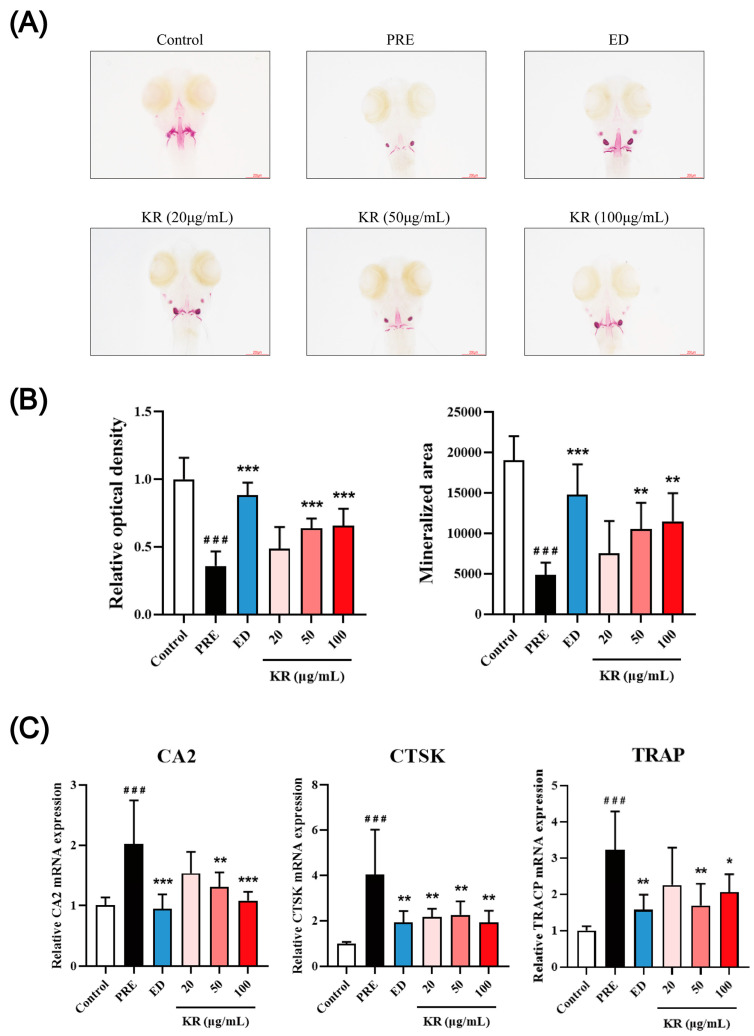
The effects of KR on bone mineralization of zebrafish larvae skull. (**A**) The dorsal aspect head bone stained with ARS in zebrafish larvae at 9 days postfertilization (dpf) with or without exposure to KR (20 μg/mL, 50 μg/mL, and 100 μg/mL). (**B**) The effect of KR on bone relative optical density and bone mineralization area in 9-dpf zebrafish. (**C**) The effect of KR on osteoclast-gene expression in 9-dpf zebrafish. ^###^ *p* < 0.001 compared with control. * *p* < 0.05; ** *p* < 0.01; *** *p* < 0.001 compared with PRE.

The network pharmacology, molecular docking, and molecular dynamics results provided certain potential critical targets of KR to alleviate OP. We further confirmed the hypothesis of those key protein targets and signaling cascades. In this study, the prednisone (PRE)-induced OP zebrafish larvae were treated to either 20, 50, or 100 μg/mL KR extract, respectively. As shown in Figure 10A,B, the bone relative optical density and bone mineralized area in the PRE-induced OP model were significantly declined compared to the control group (*p* < 0.001). Compared with the model group, 120 μM etidronate disodium (ED) (as positive control) could reverse the decreased mineralized bone area and relative optical density (*p* < 0.001) in the zebrafish model. Meanwhile, the KR extract (50 and 100 μg/mL) significantly increased the mineralized bone area and relative optical density in a dose-dependent manner in PRE-induced OP zebrafish larvae (*p* < 0.01). These results demonstrated that KR could alleviate glucocorticoid-induced cranial bone loss in the zebrafish model.

TRAP, CTSK, and CA2 are well-recognized markers of osteoclasts and osteoclastogenesis. As shown in Figure 10C, 25 μM PRE significantly increased the mRNA levels of TRAP, CTSK, and CA2 compared with the control group (*p* < 0.001). Treatment by ED and KR extract significantly decreased the induced mRNA expression of TRAP, CTSK, and CA2 by PRE (*p* < 0.05). These findings further suggested that KR could inhibit glucocorticoid-induced promotion of osteoclastic differentiation.

**Figure 11:**
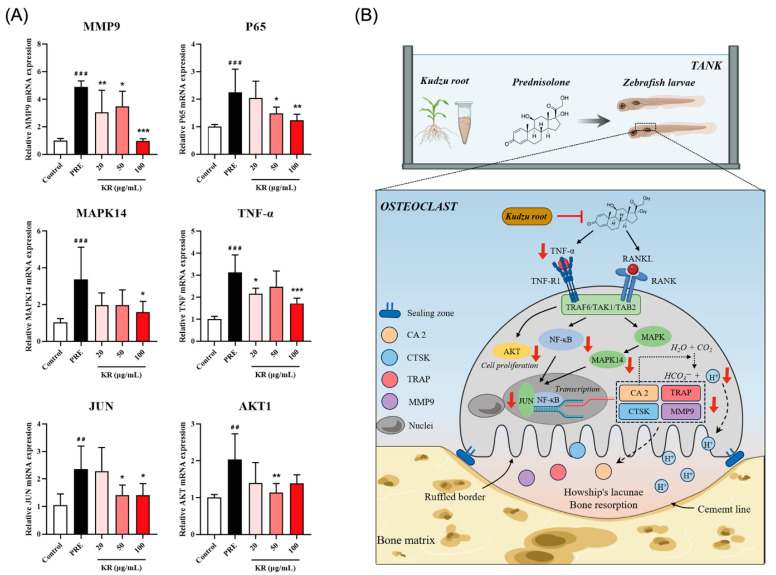
Impact of KR on the expression of osteoclastic differentiation-related targets in 9-dpf zebrafish larvae. (**A**) Changes in the relative mRNA levels of AKT1, JUN, MAPK14, MMP9, P65, and TNF-α were determined by RT-qPCR (*n* = 6). (**B**) KR and hitting targets on osteoclast differentiation pathways. ^##^ *p* < 0.01; ^###^ *p* < 0.001 compared with control. * *p* < 0.05; ** *p* < 0.01; *** *p* < 0.001 compared with PRE.

The network pharmacology and molecular docking and dynamics results indicated potential key targets and pathways for KR against OP. In this study, the experimental validation was also performed through a PRE-induced OP model in zebrafish. The mRNA expression levels of these key targets (MMP9, P65, MAPK14, TNF-α, JUN, and AKT1) in zebrafish larvae were assessed by RT-qPCR. As shown in Figure 11A, compared with the corresponding control group, the mRNA expression levels of MMP9, P65, MAPK14, TNF-α, JUN, and AKT1 were significantly up-regulated in zebrafish larvae by 25 μM PRE (*p* < 0.01). A total of 20 μg/mL KR extract could significantly reduce the PRE-induced up-regulation of MMP9 and TNF-α; 50 μg/mL KR extract inhibits the mRNA expression levels of MMP9, P65, JUN, and AKT1; and 100 μg/mL KR extract could markedly suppress the mRNA expression levels of MMP9, P65, MAPK14, TNF-α, and JUN. These results further demonstrated that KR could inhibit the PRE-induced promotion of osteoclastogenesis by down-regulating the expression of gene markers involved in the TNF-α/AKT/NF-κB/p38 MAPK signaling axis (Figure 11B), indicating that our network pharmacology-based prediction model for molecular mechanism investigation is reliable.

## 3. Materials and Methods

### 3.1. The Network Pharmacology Analysis of KR

#### 3.1.1. Data Preparation

##### Construction of the Ingredient Database for KR and Screening of Active Compounds

The compounds of KR were looked up from the TCMSP [49], TCMID [50], and ETCM [51] databases as previously described [52]. The pharmacokinetic properties ADME are considered key contributors affecting bioactivities. In this study, the screening criteria for oral bioavailability (OB) and drug-likeness (DL) were set to be greater than 30% and 0.18, respectively. Moreover, some ingredients with good bioactivities reported in other in vivo studies, which did not satisfy these two criteria, were also considered as candidate bioactive compounds for further analysis. The screening criteria for gastrointestinal absorption were set to “High” in the SwissADME database. The PubChem ID and 2D structure of the KR compounds were retrieved from PubChem [53].

##### Target Prediction of KR and Screening of OP-Related Target

The potential targets of the active compounds derived from KR for *Homo sapiens* were predicted from the SwissTargetPrediction webserver [54], DrugBank database [55], BATMAN-TCM database [56], and PubMed database as previously described [52]. The disease target information related to OP was searched for on public databases, including GeneCards [57], DisGeNET [58], DrugBank [55], TTD [59], and OMIM [60] databases. The standard gene names and UniProt ID of OP-related targets were obtained from the UniprotKB database.

##### PPI Data

After overlapping the drug- and disease-related targets, the PPI of each target was generated from the STRING database [61]. This database defines a PPI according to the interaction scores [high (>0.7), medium (>0.4), and low (>0.15)]. In this study, the medium confidence score of 0.4 was used to construct the PPI network.

#### 3.1.2. Network Construction

The Cytoscape (Version 3.7.1) software [62] was used to generate various networks, including the active compounds of a KR-predicted target network, KR anti-OP target network, and PPI network. The highly connected PPI sub-network was generated using MCODE plugin of Cytoscape, the chosen parameters for the analysis were as follows: node score cutoff equal to or greater than 0.2, K-core equal to or greater than 4, degree cutoff equal to or greater than 2, and maximum depth equal to 100 [52,63]. The node degree values in networks were calculated by the Cytoscape plugin “Network Analyzer”.

#### 3.1.3. Gene Ontology and KEGG Pathway Enrichment Analysis

The enrichment analysis of GO (BP, MF, and CC) and KEGG signaling pathways were carried out using the Metascape database [64]. We conducted the functional enrichment analysis of the gene symbols of the anti-OP targets of KR, the chosen parameters for GO and KEGG analysis were as follows: input and analysis as species = *Homo sapiens*, min overlap = 3, *p* value cutoff = 0.05, min enrichment = 1.5, the visualization analysis was carried out by RStudio-4.4.2 software.

### 3.2. Molecular Docking

The three-dimensional structures of the active ingredients of KR were prepared utilizing the ChemBio3D Ultra 14.0 software. The crystallized structures of AKT1 (PDB ID: 6HHI, Resolution: 2.70 Å, Co-crystallized ligand ID: G4N), MAPK14 (PDB ID: 6SFO, Resolution: 1.75 Å, Co-crystallized ligand ID: LEB), MMP9 (PDB ID: 6ESM, Resolution: 1.104 Å, Co-crystallized ligand ID: B9Z), TNF-α (PDB ID: 6X82, Resolution: 2.75 Å, Co-crystallized ligand ID: UTM), JUN (PDB ID: 4Y5H, Resolution: 2.055 Å, Co-crystallized ligand ID: 519), P65 (PDB ID: 7O5X, Resolution: 1.80 Å, Co-crystallized ligand ID: V3W), IL1B (PDB ID: 6Y8M, Resolution: 1.90 Å, Co-crystallized ligand ID: SX2), and IL6 (PDB ID: 1ALU, Resolution: 1.90 Å, Co-crystallized ligand ID: TLA) were downloaded from the RCSB database [65]. Using AutoDockTools-1.5.6, the crystal structures of the protein were processed by deleting water and adding hydrogen and setting the protein as a receptor; the ingredient of KR was set to a ligand. Subsequently, the ligand and receptor structures were both exported in PDBQT format. The molecular docking range was then defined by importing the receptor and ligand PDBQT formats into AutoDock4. The grid box (number of points: X = 50, Y = 50, Z = 50) of AKT1 (center_X = −2.774, center_Y = 6.152, center_Z = −10.74), MAPK14 (center_X = 0.05, center_Y = 1.072, center_Z = −19.152), MMP9 (center_X = 0.794, center_Y = 50.361, center_Z = 19.921), TNF-α (center_X = −28.569, center_Y = 3.166, center_Z = 7.208), JUN (center_X = 0.882, center_Y = −29.012, center_Z = −30.232), P65 (center_X = 24.03, center_Y = 21.694, center_Z = 0.925), IL1B (center_X = 8.066, center_Y = 25.055, center_Z = −9.87), and IL6 (center_X = −7.677, center_Y = −12.743, center_Z = 0.048) were set separately for the docking process. The receptor protein was set to rigid docking and the genetic algorithm was selected. The docking results were obtained by running Autodock Vina-1.1.2, by which the binding affinity between the protein and the compound was revealed. Finally, we used PyMOL-1.7.2.1 and Discovery studio-2020 to output the composite PDB format file visually.

### 3.3. Molecular Dynamics

Molecular dynamics of the complexes obtained from molecular docking were conducted using GROMACS 2022.3 like in previous studies [66,67,68]. Briefly, the Amber99sb-ildn force field and Tip3p water model were used in the simulation system. The total charge of the system was neutralized by adding suitable number of Na+ ions. The steepest-descent method was used for energy minimization. After energy minimization, the equilibrium simulations were performed using both isothermal isovolumic (NVT) and isothermal isobaric (NPT) ensembles. Subsequently, the molecular dynamics simulations were run for 100 ns consisting of 5 million steps with a step length of 2 fs at 300 K and 1 atmosphere of pressure. Upon completion of the simulation, the built-in tool of the software was used to analyze the trajectory. The RMSD, RMSF, hydrogen bond, and Gibbs free energy were calculated.

### 3.4. Experimental Validation

#### 3.4.1. Materials and Chemicals

KR extract containing 70% flavonoids, of which puerarin content constitutes 30% of flavonoids, was purchased from Xi’an Tonking Biotech Co., Ltd. (Xi’an, China). Dimethyl sulfoxide (DMSO) was purchased from Solarbio Life Sciences Co., Ltd. (Beijing, China). Prednisolone, Etidronate disodium, and Alizarin Red S (ARS) were purchased from Sigma-Aldrich (St. Louis, MO, USA).

#### 3.4.2. Animals

The zebrafish is an effective model for studying bone diseases due to its bone structure, metabolism, signaling pathways, and phenotypes, which closely resemble those in humans [69,70]. Additionally, the influence of genetics or chemicals on bone formation and growth in zebrafish can be quickly evaluated due to their rapid embryonic development. In this study, adult zebrafish (*Danio rerio*) were maintained in the tank flowthrough system according to standard conditions (14:10 h light/dark cycle at 28 °C). Subsequently, embryos were typically harvested from nature crosses of 4–5 pairs of adult zebrafish and kept in E3 zebrafish embryos medium (5 mM NaCl, 0.17 mM KCl, 0.33 mM CaCl_2_, 0.33 mM MgSO_4_, and 0.1% Methylene Blue) for further analysis. All animal procedures were performed in accordance with the Guidelines for Care and Use of Laboratory Animals and approved by the Animal Subjects Ethics Sub-Committee of The Hong Kong Polytechnic University (Case No. 22-23/314-ABCT-R-GRF).

#### 3.4.3. Experimental Procedures

3 days after fertilization (dpf) newly hatched zebrafish larvae were put into 24-well plates (*n* = 8 larvae/well/group) with a blank E3 medium. The zebrafish larvae were then randomly divided into six groups: 0.1% DMSO (control group), 25 μM prednisone (model group), 25 μM prednisone + 120 μM etidronate disodium (positive group), 25 μM prednisone + 20 μg/mL KR (low-dose group), 25 μM prednisone + 50 μg/mL KR (middle-dose group), and 25 μM prednisone + 100 μg/mL KR (high-dose group) from 3 dpf to 9 dpf. The dosages of prednisone and etidronate disodium are based on previous studies, and the dosage of KR was determined based on our preliminary experiments [71,72]. The 9 dpf zebrafish larvae were meticulously collected for the subsequent evaluation.

#### 3.4.4. Skeletal Staining

All zebrafish larvae were euthanized with 0.02% ethyl 3-aminobenzoate methanesulfonate (MS-222) at the 9 dpf. After removing the MS-222, zebrafish larvae were fixed in 4% paraformaldehyde for 2 h and then bleached to transparency using a decolorizer containing 3% H_2_O_2_ and 0.5% KOH for 1 h. Next, the zebrafish larvae were rinsed with 25% glycerin/0.1% KOH several times and stained with 0.1% ARS stain overnight. Subsequently, the zebrafish larvae were transparent with 50% glycerin/0.1% KOH for 10 min to clear the background and then the zebrafish larvae were stored in fresh 50% glycerin/0.1% KOH for subsequent observation. The zebrafish larval were positioned on a dish, and the images of the stained head bone were captured using stereomicroscope (Nikon, SMZ1270i, Kyoto, Japan). Bone mineralization and bone mineral density were assessed through the calculation of the mineralized bone area and relative optical density using Image J-2.1.0 analysis software.

#### 3.4.5. Real-Time PCR

The total RNA of zebrafish larvae was extracted using RNAiso Plus (TaKaRa, Kyoto, Japan) according to manufacturer’s instructions. Then, RT-qPCR was performed in QuantStudio 7 Flex Real-Time PCR System (ThermoFisher Scientific, MA, USA) using the One-Step TB Green^®^ PrimeScript™ RT-PCR Kit II (TaKaRa, Kyoto, Japan). Primers were synthesized by TechDragonLimited (Shatin, Hong Kong) and are detailed in Table S10.

### 3.5. Statistical Analysis

All data are expressed as mean ± standard deviation (SD). One-way ANOVA and *t*-tests analysis were performed using GraphPad Prism version 8.0. A *p*-value of <0.05 was considered as a significant difference.

## 4. Conclusions

In summary, our findings elucidate the cooperation among multiple components and multiple targets of KR in the treatment of OP. Specifically, we propose that AKT1, P65, MAPK14, JUN, TNF-α, and MMP9, which are involved in the TNF signaling pathway and osteoclast differentiation pathway, are the precise targets through which KR prevents OP. From the clinical perspective, in addition to the RANK/RANKL/OPG signaling pathway, the aforementioned critical targets could be considered as a new strategy for the development of drugs designed to suppress excessive osteoclast activation. Moreover, as a commonly utilized traditional medicinal food, KR could potentially be used as an appropriate clinical strategy and as part of a complementary approach in conjunction with other treatments for the management of OP, such as calcium and vitamin D supplementation and clinical anti-osteoporotic medication. Overall, our findings provide a theoretical basis and research data for the clinical application of KR in the treatment of OP and systematically reveal the pharmacodynamic basis and integrated pharmacological mechanisms of action of KR against OP.

However, our study has some limitations. Firstly, network pharmacology studies are typically based on computational predictions. Although the predicted molecular targets of KR against OP were validated at the mRNA level in this study, integrating other more direct and precise methods such as high-throughput sequencing is necessary to confirm our findings in future studies. Secondly, we only examine the effects of KR extract on the predicted targets, rather than each single compound isolated from KR. Therefore, future studies will focus on investigating the regulatory effects of the bioactive compounds isolated from KR on the predicted targets in osteoclast differentiation model in vitro and mammalian OP models in vivo. Meanwhile, we will utilize the Cellular Thermal Shift Assay (CETSA), Surface Plasmon Resonance (SPR), Isothermal Titration Calorimetry (ITC), and MicroScale Thermophoresis (MST) techniques to further verify the interaction between the bioactive compounds of KR and the predicted target proteins in future studies.

## Figures and Tables

**Table 1:** The 15 active compounds of KR.

**ID**	**Name**	**2D Structure**	**OB**	**DL**	**Gastrointestinal Absorption**
KR1	3′-Methoxydaidzein	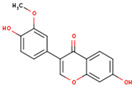	48.57	0.24	High
KR2	3′-Methoxypuerarin	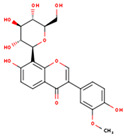	35.85	0.27	High
KR3	Coumestrol	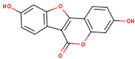	32.49	0.34	High
KR4	Daidzein	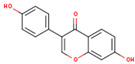	19.44	0.19	High
KR5	Daidzin	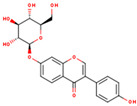	14.32	0.73	High
KR6	Dalbergenone	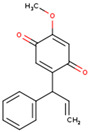	_	_	High
KR7	Formononetin	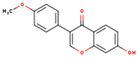	69.67	0.21	High
KR8	Formononetin-7-glucoside	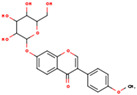	11.52	0.78	High
KR9	Genistein 7-glucoside	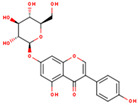	13.35	0.75	High
KR10	Genistein 8-c-glucoside	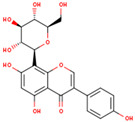	15.27	0.72	High
KR11	Genistein	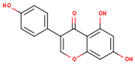	17.93	0.21	High
KR12	Methyl-p-hydroxycinnamate	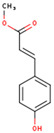	36.85	0.26	High
KR13	Puerarin	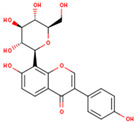	24.03	0.69	High
KR14	Puerarol	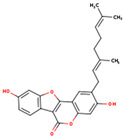	_	_	High
KR15	Scoparone	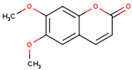	74.75	0.09	High

**Table 2:** Annotation of KEGG pathways.

Term ID	Description	Count	*p* Value	Genes
hsa05200	Pathways in cancer	59	2.56 × 10^−33^	AGTR1, AKT1, AR, BAD, CCND1, BCL2, CALM1, CSF1R, ESR1, ESR2, F2, FLT3, FOS, HMOX1, IGF1R, IL2, IL6, JAK2, JUN, KIT, MMP2, MMP9, NFKB1, NOS2, PDGFRA, PDGFRB, PPARG, PRKACA, MAPK1, MAPK3, PTGS2, RAD51, RELA, RXRA, TERT, TP53, VEGFA, MAPK14, SRC, GHR, IFNB1, INS, NOS3, IL1B, TNF, LDLR, PPARA, IGFBP3, HTR2A, HTR2C, CAT, ABCB1, ADRB2, KCNJ11, KCNN3, PDE4D, MTNR1B, CRHR1 ICAM1
hsa05417	Lipid and atherosclerosis	63	1.58 × 10^−32^	AKT1, BAD, BCL2, CALM1, MAPK14, CYP2B6, FOS, ICAM1, IFNB1, IL1B, IL6, JAK2, JUN, LDLR, MMP9, NFKB1, NOS3, PPARG, MAPK1, MAPK3, RELA, RXRA, CCL2, SELE, SRC, TNF, TP53, VCAM1, AGTR1, CCND1, MMP2, VEGFA, NOX4, HMOX1, PTGS2, IL2, NOS2, TLR9, MALT1, PDGFRA, PRKACA, ESR1, ESR2, INS, CTSK, CSF1R, CAT, CYP1B1, SOD1, PARP1, TERT, F2, PPARA, ALOX12, ABCC1, ABCG2, ALDH2, P2RX7, APP, IGFBP3, CXCR3, ARG1, ELANE
hsa05207	Chemical carcinogenesis—receptor activation	27	7.11 × 10^−25^	ADRB2, AKT1, AR, BAD, CCND1, BCL2, CYP1B1, CYP2B6, CYP3A4, ESR1, ESR2, FOS, JAK2, JUN, NFKB1, PPARA, PRKACA, MAPK1, MAPK3, RELA, RXRA, SRC, VEGFA, CALM1, CFTR, CRHR1, PDE4D
hsa04668	TNF signaling pathway	18	1.63 × 10^−22^	AKT1, MAPK14, FOS, ICAM1, IFNB1, IL1B, IL6, JUN, MMP9, NFKB1, MAPK1, MAPK3, PTGS2, RELA, CCL2, SELE, TNF, VCAM1
hsa04380	Osteoclast differentiation	14	1.84 × 10^−15^	AKT1, MAPK14, CSF1R, CTSK, FOS, IFNB1, IL1B, JUN, NFKB1, PPARG, MAPK1, MAPK3, RELA, TNF
hsa05202	Transcriptional misregulation in cancer	13	5.94 × 10^−12^	CSF1R, ELANE, FLT3, HPGD, IGF1R, IGFBP3, IL6, MMP9, NFKB1, PPARG, RELA, RXRA, TP53
hsa05206	MicroRNAs in cancer	19	1.15 × 10^−11^	CCND1, BCL2, CYP1B1, HMOX1, MMP9, ABCC1, NFKB1, PDGFRA, PDGFRB, ABCB1, MAPK1, MAPK3, PTGS2, TP53, VEGFA, AKT1, NOS2, RELA, RXRA
hsa04064	NF-kappa B signaling pathway	13	6.71 × 10^−11^	PARP1, BCL2, ICAM1, IL1B, NFKB1, PTGS2, RELA, TNF, VCAM1, MALT1, ALOX15, IFNB1, JAK2
hsa05144	Malaria	12	1.15 × 10^−10^	ICAM1, IL1B, IL6, CCL2, SELE, TNF, VCAM1, TLR9, CTSK, FOS, JUN, VEGFA
hsa04913	Ovarian steroidogenesis	8	1.32 × 10^−10^	CYP1B1, CYP19A1, HSD17B1, IGF1R, INS, LDLR, PRKACA, PTGS2
hsa05415	Diabetic cardiomyopathy	14	1.54 × 10^−10^	PARP1, AGTR1, AKT1, MAPK14, GSR, INS, MMP2, MMP9, NFKB1, NOS3, PPARA, RELA, ICAM1, VCAM1
hsa04726	Serotonergic synapse	11	1.56 × 10^−10^	ALOX12, ALOX15, APP, HTR2A, HTR2C, PRKACA, MAPK1, MAPK3, PTGS2, SLC6A4, CYP2B6
hsa04211	Longevity regulating pathway	13	3.71 × 10^−10^	AKT1, CAT, IGF1R INS, NFKB1, PPARG, PRKACA, RELA, TP53, SOD1, CCND1, CFTR, HMGCR
hsa04976	Bile secretion	10	3.71 × 10^−10^	CA2, CFTR, CYP3A4, HMGCR, LDLR, ABCB1, PRKACA, RXRA, ABCG2, ABCC1
hsa00140	Steroid hormone biosynthesis	10	4.92 × 10^−10^	CYP1B1, CYP3A4, CYP11B1, CYP11B2, CYP19A1, HSD11B1, HSD17B1, SRD5A1, PTGS2, CYP2B6
hsa04020	Calcium signaling pathway	17	9.02 × 10^−10^	ADRB2, AGTR1, CALM1, HTR2A, HTR2C, NOS2, NOS3, P2RX7, PDGFRA, PDGFRB, PRKACA, VEGFA, CRHR1, F2, GHR, GPR35, MTNR1B
hsa04630	JAK-STAT signaling pathway	10	3.78 × 10^−9^	AKT1, CCND1, BCL2, GHR, IFNB1, IL2, IL6, JAK2, PDGFRA, PDGFRB
hsa04750	Inflammatory mediator regulation of TRP channels	18	1.96 × 10^−8^	ALOX12, CALM1, MAPK14, HTR2A, HTR2C, IL1B, PRKACA, SRC, ADRB2, AGTR1, FOS, JUN, AKT1, CA2, CFTR, PPARA, SOD1, TP53
hsa04923	Regulation of lipolysis in adipocytes	10	2.84 × 10^−7^	ADRB2, AKT1, INS, PRKACA, PTGS2, MGLL, MAPK14, MAPK1, MAPK3, PPARG
hsa04060	Cytokine-cytokine receptor interaction	12	7.10 × 10^−6^	CSF1R, GHR, CXCR3, IFNB1, IL1B, IL2, IL6, CCL2, TNF, FLT3, KIT, INS

**Table 3:** Free binding energy of AKT1, JUN, MAPK14, MMP9, P65, TNF-α, IL1B, and IL6 with their corresponding active compounds.

Target	Compound	Free Bind Energy (kcal/mol)
AKT1	KR3	−10.4
JUN	KR4	−7.2
	KR7	−7.7
	KR11	−7.7
	KR13	−7.8
MAPK14	KR4	−9.2
	KR6	−8.2
	KR7	−9.3
	KR8	−8.4
	KR11	−8.5
	KR13	−9.1
MMP9	KR3	−8.7
	KR11	−11.3
	KR13	−7.9
P65	KR11	−7.2
	KR13	−7.8
	KR15	−5.4
TNF-α	KR4	−9.0
	KR5	−9.4
	KR8	−9.3
	KR9	−10.2
	KR11	−9.7
	KR13	−9.4
IL1B	KR11	−4.8
IL6	KR4	−5.4

**Table 4:** MM/GBSA results of the Coumestrol-AKT1, Puerarin-JUN, Formononetin-MAPK14, Genistein-MMP9, Puerarin-P65, and Genistein 7-glucoside-TNF-α complexes.

Energy Component	Coumestrol-AKT1	Puerarin-JUN	Formononetin-MAPK14	Genistein-MMP9	Puerarin-P65	Genistein 7-Glucoside-TNF-α
ΔVDWAALS	−26.29 ± 1.95	−36.83 ± 1.95	−27.92 ± 1.79	−41.96 ± 1.80	−25.88 ± 3.34	−44.63 ± 2.49
ΔEEL	−29.45 ± 5.89	−11.31 ± 5.07	−15.25 ± 5.46	−0.41 ± 3.58	−63.60 ± 6.37	−31.24 ± 6.25
ΔEGB	32.34 ± 3.34	23.32 ± 3.58	20.96 ± 3.24	18.33 ± 2.66	63.75 ± 3.76	42.06 ± 3.49
ΔESURF	−4.42 ± 0.18	−4.75 ± 0.18	−4.76 ± 0.17	−4.72 ± 0.06	−5.12 ± 0.20	−6.65 ± 0.14
ΔGGAS	−55.73 ± 4.52	−48.14 ± 5.71	−43.16 ± 5.83	−42.37 ± 3.27	−89.48 ± 6.42	−75.87 ± 6.77
ΔGSOLV	27.92 ± 3.32	18.57 ± 3.62	16.20 ± 3.16	13.61 ± 2.67	58.63 ± 3.72	35.41 ± 3.41
ΔTOTAL	−27.82 ± 1.82	−29.57 ± 3.34	−26.96 ± 3.14	−28.76 ± 1.51	−30.85 ± 3.85	−40.46 ± 3.41

Notes: ΔVDWAALS: Van der Waals energy; ΔEEL: electrostatic energy; ΔEGB: polar solvation energy; ΔESURF: non-polar solvation energy; ΔGGAS: gas-phase molecular mechanics free energy; ΔGSOLV: solvation free energy; ΔTOTAL: combined total energy.

## Data Availability

All data are available on request from the authors.

## Supplementary Materials

The following supporting information can be downloaded at: https://www.mdpi.com/article/10.3390/ijms26031202/s1.

## Abbreviations

ADME, absorption distribution metabolism and excretion; ARS, alizarin red S; BP, biological processes; CA2, carbonic anhydrase II; CC, cellular component; CTSK, cathepsin K; DPF, days after fertilization; ED, etidronate disodium; GO, gene ontology; IL1B, interleukin 1 Beta; IL6, interleukin 6; MAPKs, mitogen-activated protein kinases; MF, molecular function; MMP9, matrix metalloproteinase 9; NFATc1, nuclear factor of activated T-cell cytoplasmic 1; OP, osteoporosis; OVX, ovariectomy; KR, Kudzu root; PPI, protein-protein interaction; RANK, receptor activator of nuclear factor κB; TCM, Traditional Chinese Medicine; TNF-α, tumour necrosis factor-α; TRAP, Tartrate-resistant acid phosphatase; TRAF6, TNF receptor associated factor 6.
