# Supplementary material for: Network Pharmacology Analysis and Biological Validation Systemically Identified the Active Ingredients and Molecular Targets of Kudzu Root on Osteoporosis
Source: Int J Mol Sci. 2025 Jan 30;26(3):1202. doi: 10.3390/ijms26031202 (PMC11818621; doi:10.3390/ijms26031202)
Supplement: Supplementary file 1 [file ijms-26-01202-s001.zip › ijms-3414783-supplementary.pdf]

Supplementary materials

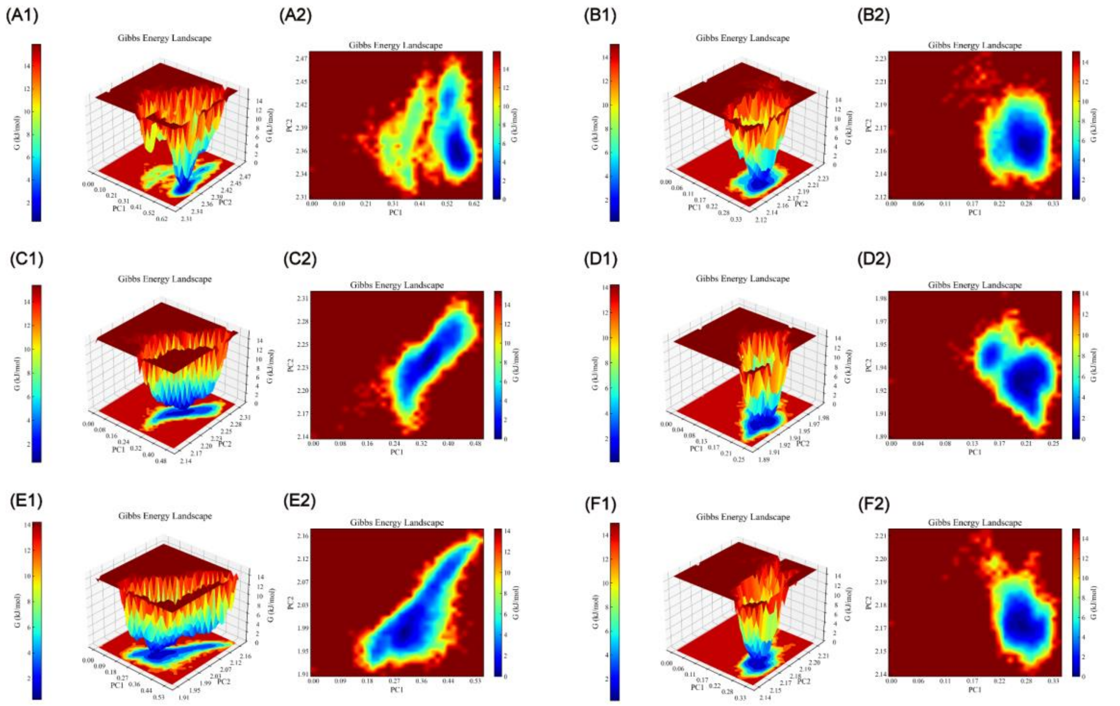

Figure S1. 3D and 2D Gibbs free energy landscape of the Coumestrol-AKT1 **(A1-A2)**, Puerarin-JUN **(B1-B2)**, Formononetin-MAPK14 **(C1-C2)**, Genistein-MMP9 **(D1-D2)**, Puerarin-P65 **(E1-E2)**, and Genistein 7-glucoside-TNF-α **(F1-F2)** complexes.

Table S1. Data on the Kudzu root compound–predicted target network.

Name	Degree	Betweenness Centrality	Closeness Centrality	Average Shortest Path Length
KR11	124	0.3202	0.4696	2.1296
KR6	101	0.4251	0.4381	2.2824
KR7	84	0.1403	0.4175	2.3953
KR3	84	0.2184	0.4175	2.3953
KR4	82	0.1288	0.4152	2.4086
KR1	47	0.0300	0.3786	2.6412
KR13	34	0.0640	0.3666	2.7276
KR8	32	0.0549	0.3648	2.7409
KR15	25	0.0317	0.3588	2.7874
KR5	25	0.0173	0.3588	2.7874
KR14	19	0.0619	0.3537	2.8272
KR9	15	0.0034	0.3504	2.8538
CA12	15	0.0564	0.5128	1.9502
CA7	13	0.0210	0.4118	2.4286
KR12	12	0.0017	0.3480	2.8738
PTGS2	11	0.0335	0.4894	2.0432
CA2	11	0.0106	0.3976	2.5150
CA1	10	0.0134	0.3987	2.5083
CA9	10	0.0401	0.4910	2.0365

KR2	9	0.0049	0.3456	2.8937
KR10	8	0.0042	0.3448	2.9003
AKR1B1	8	0.0052	0.3640	2.7475
AR	8	0.0050	0.3605	2.7741
XDH	8	0.0056	0.3834	2.6080
ESR1	8	0.0080	0.3935	2.5415
CA13	7	0.0095	0.3864	2.5880
PTPN1	7	0.0163	0.4319	2.3156
CALM1	7	0.0028	0.3326	3.0066
PIM1	7	0.0142	0.4210	2.3754
HSP90AA1	7	0.0088	0.3854	2.5947
PPARG	7	0.0051	0.3825	2.6146
NOS2	7	0.0087	0.3748	2.6678
ADORA1	7	0.0028	0.3588	2.7874
MAOA	7	0.0176	0.4568	2.1894
ALDH2	7	0.0028	0.3588	2.7874
ESR2	7	0.0168	0.4540	2.2027
CA4	7	0.0043	0.3767	2.6545
F2	6	0.0029	0.3178	3.1462
TNF	6	0.0032	0.3545	2.8206
IL2	6	0.0025	0.3571	2.8007
PRKACA	6	0.0021	0.3545	2.8206

GSK3B	6	0.0172	0.4540	2.2027
MAPK14	6	0.0140	0.4319	2.3156
MAOB	6	0.0123	0.4245	2.3555
EGFR	6	0.0156	0.4526	2.2093
CA5A	5	0.0027	0.3666	2.7276
CA14	5	0.0027	0.3666	2.7276
CA6	5	0.0027	0.3666	2.7276
TNKS	5	0.0144	0.4499	2.2226
TNKS2	5	0.0144	0.4499	2.2226
ACHE	5	0.0028	0.3730	2.6811
PTGS1	5	0.0016	0.3504	2.8538
ADORA2A	5	0.0097	0.4186	2.3887
HTR2C	5	0.0097	0.4186	2.3887
TBXAS1	5	0.0097	0.4186	2.3887
ALOX12	5	0.0025	0.3711	2.6944
TLR9	5	0.0097	0.4186	2.3887
PTPRS	5	0.0025	0.3711	2.6944
CBR1	5	0.0025	0.3711	2.6944
HSD17B2	5	0.0025	0.3711	2.6944
TYR	5	0.0025	0.3711	2.6944
JUN	4	0.0020	0.3545	2.8206
CA5B	4	0.0006	0.3139	3.1860

SLC6A2	4	0.0086	0.4163	2.4020
PFKFB3	4	0.0021	0.3693	2.7076
NOX4	4	0.0021	0.3693	2.7076
ALOX5	4	0.0115	0.4343	2.3023
SLC29A1	4	0.0006	0.2816	3.5515
PRSS1	4	0.0007	0.3464	2.8870
CHEK1	4	0.0007	0.3464	2.8870
ABCG2	4	0.0007	0.3464	2.8870
ESRRB	4	0.0007	0.3464	2.8870
ESRRA	4	0.0007	0.3464	2.8870
HTR2A	4	0.0007	0.3464	2.8870
MGAM	4	0.0007	0.3464	2.8870
HSD17B1	4	0.0007	0.3464	2.8870
PPARA	4	0.0007	0.3464	2.8870
ABCB1	4	0.0007	0.3464	2.8870
CYP19A1	4	0.0007	0.3464	2.8870
MIF	4	0.0007	0.3464	2.8870
RELA	3	0.0013	0.3363	2.9734
CHRM1	3	0.0038	0.3562	2.8073
SLC6A4	3	0.0038	0.3562	2.8073
F7	3	0.0001	0.2714	3.6844
DPP4	3	0.0004	0.3050	3.2791

ADORA3	3	0.0043	0.3275	3.0532
VCAM1	3	0.0012	0.3417	2.9269
VEGFA	3	0.0012	0.3417	2.9269
ADRB2	3	0.0005	0.3126	3.1993
F10	3	0.0027	0.3417	2.9269
PLAT	3	0.0012	0.3417	2.9269
STS	3	0.0005	0.3448	2.9003
PON1	3	0.0005	0.3448	2.9003
ALOX15	3	0.0005	0.3448	2.9003
TOP1	3	0.0018	0.3537	2.8272
MMP2	3	0.0021	0.3537	2.8272
MMP9	3	0.0021	0.3537	2.8272
CDK5R1	3	0.0013	0.3562	2.8073
CCNB3	3	0.0085	0.4152	2.4086
FLT3	3	0.0085	0.4152	2.4086
PARP1	3	0.0085	0.4152	2.4086
KDR	3	0.0009	0.3087	3.2392
RXRA	3	0.0001	0.3094	3.2326
AGTR1	2	0.0007	0.2764	3.6179
NFKBIA	2	0.0003	0.2790	3.5847
CCL2	2	0.0004	0.3275	3.0532
FOS	2	0.0006	0.3290	3.0399

BCL2	2	0.0006	0.3290	3.0399
AKR1B10	2	0.0006	0.3290	3.0399
PKIA	2	0.0002	0.2989	3.3455
CA3	2	0.0003	0.3363	2.9734
SELE	2	0.0038	0.3834	2.6080
JAK3	2	0.0026	0.3233	3.0930
MAPK9	2	0.0026	0.3233	3.0930
HDAC6	2	0.0038	0.3834	2.6080
SLC6A3	2	0.0020	0.3504	2.8538
MT-ND6	2	0.0001	0.3081	3.2458
ATP5F1B	2	0.0001	0.3081	3.2458
LDLR	2	0.0002	0.3326	3.0066
TP53	2	0.0002	0.3326	3.0066
PDE3A	2	0.0001	0.3081	3.2458
MCL1	2	0.0002	0.3326	3.0066
PLAU	2	0.0002	0.3326	3.0066
CYP3A4	2	0.0005	0.3304	3.0266
PIK3CG	2	0.0006	0.3432	2.9136
CRHR1	2	0.0006	0.3432	2.9136
AHR	2	0.0005	0.3304	3.0266
CDK2	2	0.0042	0.3554	2.8140
ARG1	2	0.0006	0.3432	2.9136

CD38	2	0.0006	0.3432	2.9136
MMP12	2	0.0006	0.3432	2.9136
APP	2	0.0006	0.3432	2.9136
GLO1	2	0.0006	0.3432	2.9136
NUAK1	2	0.0042	0.3554	2.8140
BACE1	2	0.0005	0.3304	3.0266
SRC	2	0.0006	0.3068	3.2591
CFTR	2	0.0006	0.3432	2.9136
TTR	2	0.0006	0.3432	2.9136
ABCC1	2	0.0006	0.3432	2.9136
SYK	2	0.0006	0.3432	2.9136
CDK6	2	0.0006	0.3432	2.9136
PLK1	2	0.0006	0.3432	2.9136
AURKB	2	0.0006	0.3432	2.9136
CSNK2A1	2	0.0006	0.3432	2.9136
TOP2A	2	0.0000	0.2575	3.8837
CCNA2	2	0.0000	0.2960	3.3787
GRIA2	1	0.0000	0.2643	3.7841
LTA4H	1	0.0000	0.2643	3.7841
GABRA1	1	0.0000	0.2643	3.7841
KLKB1	1	0.0000	0.2615	3.8239
HSP90AB1	1	0.0000	0.2615	3.8239

ACP1	1	0.0000	0.2615	3.8239
PDE4D	1	0.0000	0.2615	3.8239
CCR4	1	0.0000	0.2615	3.8239
PDK1	1	0.0000	0.2615	3.8239
HSP90B1	1	0.0000	0.2615	3.8239
SRD5A1	1	0.0000	0.2615	3.8239
MELK	1	0.0000	0.2615	3.8239
GPT	1	0.0000	0.2685	3.7243
ACE2	1	0.0000	0.2685	3.7243
IFNB1	1	0.0000	0.2685	3.7243
SELP	1	0.0000	0.2685	3.7243
GSTP1	1	0.0000	0.2685	3.7243
SOD1	1	0.0000	0.2685	3.7243
GLB1	1	0.0000	0.3199	3.1262
PTGFR	1	0.0000	0.3199	3.1262
KCNJ11	1	0.0000	0.3199	3.1262
GCKR	1	0.0000	0.3199	3.1262
INS	1	0.0000	0.3199	3.1262
HPGD	1	0.0000	0.3199	3.1262
BTK	1	0.0000	0.3199	3.1262
HMGCR	1	0.0000	0.3199	3.1262
SULT1E1	1	0.0000	0.3199	3.1262

IL1B	1	0.0000	0.3199	3.1262
CDK1	1	0.0000	0.3199	3.1262
MAPK1	1	0.0000	0.3199	3.1262
MAPK3	1	0.0000	0.3199	3.1262
BAD	1	0.0000	0.3199	3.1262
IGFBP1	1	0.0000	0.3199	3.1262
IGFBP2	1	0.0000	0.3199	3.1262
IGFBP5	1	0.0000	0.3199	3.1262
IGFBP4	1	0.0000	0.3199	3.1262
IGFBP6	1	0.0000	0.3199	3.1262
SNCA	1	0.0000	0.3199	3.1262
IGFBP3	1	0.0000	0.3199	3.1262
CYP1B1	1	0.0000	0.3199	3.1262
PDGFRA	1	0.0000	0.2676	3.7375
KIT	1	0.0000	0.2676	3.7375
ABL1	1	0.0000	0.2676	3.7375
GBA2	1	0.0000	0.2676	3.7375
MGMT	1	0.0000	0.2676	3.7375
GBA	1	0.0000	0.2676	3.7375
MBLAC2	1	0.0000	0.2948	3.3920
NOS3	1	0.0000	0.2948	3.3920
ADRA1A	1	0.0000	0.2948	3.3920

RAF1	1	0.0000	0.2948	3.3920
FEN1	1	0.0000	0.2948	3.3920
ERCC5	1	0.0000	0.2948	3.3920
DHODH	1	0.0000	0.2948	3.3920
KCNJ6	1	0.0000	0.3050	3.2791
KCNJ3	1	0.0000	0.3050	3.2791
KCNJ5	1	0.0000	0.3050	3.2791
DUSP3	1	0.0000	0.3050	3.2791
ICAM1	1	0.0000	0.3050	3.2791
IRAK4	1	0.0000	0.3050	3.2791
S1PR2	1	0.0000	0.3050	3.2791
CHRM2	1	0.0000	0.3050	3.2791
CDK7	1	0.0000	0.3050	3.2791
PARP2	1	0.0000	0.3050	3.2791
KCNN3	1	0.0000	0.3050	3.2791
GABRA2	1	0.0000	0.3050	3.2791
GABRG2	1	0.0000	0.3050	3.2791
GABRB3	1	0.0000	0.3050	3.2791
PIK3CA	1	0.0000	0.3050	3.2791
TRPA1	1	0.0000	0.3050	3.2791
TYK2	1	0.0000	0.3050	3.2791
JAK2	1	0.0000	0.3050	3.2791

JAK1	1	0.0000	0.3050	3.2791
PGGT1B	1	0.0000	0.3050	3.2791
FNTA	1	0.0000	0.3050	3.2791
PSEN2	1	0.0000	0.3050	3.2791
MAP2K1	1	0.0000	0.3050	3.2791
CES2	1	0.0000	0.3050	3.2791
CES1	1	0.0000	0.3050	3.2791
KDM4C	1	0.0000	0.3050	3.2791
SPHK1	1	0.0000	0.3050	3.2791
MGLL	1	0.0000	0.3050	3.2791
SPHK2	1	0.0000	0.3050	3.2791
ELANE	1	0.0000	0.3050	3.2791
NAMPT	1	0.0000	0.3050	3.2791
PDE10A	1	0.0000	0.3050	3.2791
MAPK10	1	0.0000	0.3050	3.2791
HTR2B	1	0.0000	0.3050	3.2791
HCRTR1	1	0.0000	0.3050	3.2791
HCRTR2	1	0.0000	0.3050	3.2791
METAP1	1	0.0000	0.3050	3.2791
CXCR3	1	0.0000	0.3050	3.2791
CCNE1	1	0.0000	0.3050	3.2791
FAP	1	0.0000	0.3050	3.2791

SIRT2	1	0.0000	0.3050	3.2791
NAAA	1	0.0000	0.3050	3.2791
PREP	1	0.0000	0.3050	3.2791
GLP1R	1	0.0000	0.3050	3.2791
HSD11B1	1	0.0000	0.3050	3.2791
CAPN1	1	0.0000	0.3050	3.2791
HMOX1	1	0.0000	0.3050	3.2791
MALT1	1	0.0000	0.3050	3.2791
PYGM	1	0.0000	0.3050	3.2791
PYGL	1	0.0000	0.3050	3.2791
CSF1R	1	0.0000	0.3050	3.2791
CCNC	1	0.0000	0.3050	3.2791
GSK3A	1	0.0000	0.3050	3.2791
NR1D1	1	0.0000	0.3050	3.2791
MTNR1B	1	0.0000	0.3050	3.2791
FABP3	1	0.0000	0.3050	3.2791
FABP4	1	0.0000	0.3050	3.2791
CTSS	1	0.0000	0.3050	3.2791
CTSK	1	0.0000	0.3050	3.2791
GABRA5	1	0.0000	0.3050	3.2791
P2RX7	1	0.0000	0.3050	3.2791
MAPK8	1	0.0000	0.3050	3.2791

ALDH1A1	1	0.0000	0.3050	3.2791
XIAP	1	0.0000	0.3050	3.2791
MTNR1A	1	0.0000	0.3050	3.2791
TGM2	1	0.0000	0.3050	3.2791
GRM5	1	0.0000	0.3050	3.2791
CYP11B2	1	0.0000	0.3050	3.2791
CYP11B1	1	0.0000	0.3050	3.2791
PRKCA	1	0.0000	0.2643	3.7841
RAD51	1	0.0000	0.2937	3.4053
LARS1	1	0.0000	0.2937	3.4053
P4HB	1	0.0000	0.2937	3.4053
GHR	1	0.0000	0.2937	3.4053
B4GALT4	1	0.0000	0.2937	3.4053
ECE1	1	0.0000	0.2937	3.4053
CAT	1	0.0000	0.2937	3.4053
IL6	1	0.0000	0.2937	3.4053
CYP2B6	1	0.0000	0.2948	3.3920
NR1I2	1	0.0000	0.2948	3.3920
TERT	1	0.0000	0.2948	3.3920
DRD3	1	0.0000	0.2948	3.3920
PTP4A3	1	0.0000	0.2948	3.3920
AKT1	1	0.0000	0.2948	3.3920

PTK2	1	0.0000	0.2948	3.3920
GPR35	1	0.0000	0.2948	3.3920
GSR	1	0.0000	0.2948	3.3920
NFKB1	1	0.0000	0.2948	3.3920
LYN	1	0.0000	0.2948	3.3920
FGR	1	0.0000	0.2948	3.3920
SQLE	1	0.0000	0.2948	3.3920
EPHB4	1	0.0000	0.2948	3.3920
MAP3K8	1	0.0000	0.2948	3.3920
AURKA	1	0.0000	0.2948	3.3920
TEK	1	0.0000	0.2948	3.3920
PLK4	1	0.0000	0.2948	3.3920
CCNA1	1	0.0000	0.2948	3.3920
FLT4	1	0.0000	0.2948	3.3920
PDGFRB	1	0.0000	0.2948	3.3920
CCND1	1	0.0000	0.2948	3.3920
MET	1	0.0000	0.2948	3.3920
IGF1R	1	0.0000	0.2948	3.3920
NCOA1	1	0.0000	0.2749	3.6379

Table S2. Data on the compound–target network of Kudzu root in osteoporosis treatment.

Name	Degree	Betweenness Centrality	Closeness Centrality	Average Shortest Path Length
KR11	65	0.4444	0.5216	1.9173
KR4	41	0.1562	0.4361	2.2932
KR7	36	0.1250	0.4249	2.3534
KR3	31	0.1847	0.4018	2.4887
KR6	28	0.2716	0.3923	2.5489
KR1	24	0.0314	0.3947	2.5338
KR13	19	0.0752	0.3833	2.6090
KR8	17	0.0636	0.3789	2.6391
KR5	12	0.0092	0.3684	2.7143
CA2	11	0.0430	0.4419	2.2632
PTGS2	11	0.0969	0.5057	1.9774
ESR1	8	0.0232	0.4332	2.3083
AR	8	0.0285	0.3923	2.5489
ESR2	7	0.0517	0.4667	2.1429
ALDH2	7	0.0083	0.3947	2.5338
NOS2	7	0.0320	0.4092	2.4436
PPARG	7	0.0165	0.4196	2.3835
CALM1	7	0.0065	0.3473	2.8797
KR9	7	0.0029	0.3566	2.8045

KR15	7	0.0064	0.3528	2.8346
MAPK14	6	0.0375	0.4419	2.2632
PRKACA	6	0.0059	0.3833	2.6090
IL2	6	0.0075	0.3923	2.5489
TNF	6	0.0091	0.3923	2.5489
F2	6	0.0105	0.3220	3.1053
KR14	6	0.0321	0.3016	3.3158
PTPRS	5	0.0095	0.4067	2.4586
TLR9	5	0.0274	0.4277	2.3383
ALOX12	5	0.0095	0.4067	2.4586
HTR2C	5	0.0274	0.4277	2.3383
MIF	4	0.0022	0.3789	2.6391
CYP19A1	4	0.0022	0.3789	2.6391
ABCB1	4	0.0022	0.3789	2.6391
PPARA	4	0.0022	0.3789	2.6391
HSD17B1	4	0.0022	0.3789	2.6391
HTR2A	4	0.0022	0.3789	2.6391
ESRRA	4	0.0022	0.3789	2.6391
ABCG2	4	0.0022	0.3789	2.6391
NOX4	4	0.0083	0.4043	2.4737
JUN	4	0.0051	0.3878	2.5789
RXRA	3	0.0003	0.3189	3.1353

PARP1	3	0.0228	0.4169	2.3985
FLT3	3	0.0228	0.4169	2.3985
MMP9	3	0.0060	0.3811	2.6241
MMP2	3	0.0060	0.3811	2.6241
ALOX15	3	0.0019	0.3768	2.6541
PON1	3	0.0019	0.3768	2.6541
F10	3	0.0135	0.3725	2.6842
ADRB2	3	0.0012	0.3220	3.1053
VEGFA	3	0.0031	0.3725	2.6842
VCAM1	3	0.0031	0.3725	2.6842
DPP4	3	0.0007	0.3086	3.2406
SLC6A4	3	0.0075	0.3384	2.9549
RELA	3	0.0032	0.3624	2.7594
KR2	2	0.0001	0.3100	3.2256
ABCC1	2	0.0024	0.3684	2.7143
TTR	2	0.0024	0.3684	2.7143
CFTR	2	0.0024	0.3684	2.7143
SRC	2	0.0018	0.3100	3.2256
APP	2	0.0024	0.3684	2.7143
MMP12	2	0.0024	0.3684	2.7143
ARG1	2	0.0024	0.3684	2.7143
CRHR1	2	0.0024	0.3684	2.7143

CYP3A4	2	0.0021	0.3402	2.9398
TP53	2	0.0008	0.3624	2.7594
LDLR	2	0.0008	0.3624	2.7594
SELE	2	0.0110	0.3855	2.5940
BCL2	2	0.0014	0.3547	2.8195
FOS	2	0.0014	0.3547	2.8195
CCL2	2	0.0012	0.3509	2.8496
KR12	2	0.0002	0.3402	2.9398
AGTR1	2	0.0019	0.2836	3.5263
IGF1R	1	0.0000	0.2873	3.4812
CCND1	1	0.0000	0.2873	3.4812
PDGFRB	1	0.0000	0.2873	3.4812
NFKB1	1	0.0000	0.2873	3.4812
GSR	1	0.0000	0.2873	3.4812
GPR35	1	0.0000	0.2873	3.4812
AKT1	1	0.0000	0.2873	3.4812
TERT	1	0.0000	0.2873	3.4812
NR1I2	1	0.0000	0.2873	3.4812
CYP2B6	1	0.0000	0.2873	3.4812
IL6	1	0.0000	0.3043	3.2857
CAT	1	0.0000	0.3043	3.2857
GHR	1	0.0000	0.3043	3.2857

P4HB	1	0.0000	0.3043	3.2857
RAD51	1	0.0000	0.3043	3.2857
CYP11B1	1	0.0000	0.2824	3.5414
CYP11B2	1	0.0000	0.2824	3.5414
TGM2	1	0.0000	0.2824	3.5414
P2RX7	1	0.0000	0.2824	3.5414
CTSK	1	0.0000	0.2824	3.5414
FABP3	1	0.0000	0.2824	3.5414
MTNR1B	1	0.0000	0.2824	3.5414
CSF1R	1	0.0000	0.2824	3.5414
MALT1	1	0.0000	0.2824	3.5414
HMOX1	1	0.0000	0.2824	3.5414
HSD11B1	1	0.0000	0.2824	3.5414
PREP	1	0.0000	0.2824	3.5414
SIRT2	1	0.0000	0.2824	3.5414
CXCR3	1	0.0000	0.2824	3.5414
ELANE	1	0.0000	0.2824	3.5414
MGLL	1	0.0000	0.2824	3.5414
JAK2	1	0.0000	0.2824	3.5414
KCNN3	1	0.0000	0.2824	3.5414
ICAM1	1	0.0000	0.2824	3.5414
ERCC5	1	0.0000	0.2989	3.3459

FEN1	1	0.0000	0.2989	3.3459
NOS3	1	0.0000	0.2989	3.3459
GBA	1	0.0000	0.2754	3.6316
KIT	1	0.0000	0.2754	3.6316
PDGFRA	1	0.0000	0.2754	3.6316
KR10	1	0.0000	0.2824	3.5414
CYP1B1	1	0.0000	0.3437	2.9098
IGFBP3	1	0.0000	0.3437	2.9098
IGFBP6	1	0.0000	0.3437	2.9098
IGFBP4	1	0.0000	0.3437	2.9098
IGFBP5	1	0.0000	0.3437	2.9098
IGFBP2	1	0.0000	0.3437	2.9098
IGFBP1	1	0.0000	0.3437	2.9098
BAD	1	0.0000	0.3437	2.9098
MAPK3	1	0.0000	0.3437	2.9098
MAPK1	1	0.0000	0.3437	2.9098
IL1B	1	0.0000	0.3437	2.9098
HMGCR	1	0.0000	0.3437	2.9098
HPGD	1	0.0000	0.3437	2.9098
INS	1	0.0000	0.3437	2.9098
KCNJ11	1	0.0000	0.3437	2.9098
GLB1	1	0.0000	0.3437	2.9098

SOD1	1	0.0000	0.2777	3.6015
IFNB1	1	0.0000	0.2777	3.6015
GPT	1	0.0000	0.2777	3.6015
SRD5A1	1	0.0000	0.2321	4.3083
PDE4D	1	0.0000	0.2321	4.3083

Table S3. Data on PPI network.

Name	Degree	Betweenness Centrality	Closeness Centrality	Average Shortest Path Length
IL6	80	0.0592	0.7548	1.3248
AKT1	80	0.0668	0.7548	1.3248
INS	79	0.0919	0.7500	1.3333
TNF	76	0.0473	0.7358	1.3590
TP53	68	0.0650	0.7048	1.4188
VEGFA	67	0.0239	0.6964	1.4359
IL1B	65	0.0259	0.6882	1.4530
SRC	63	0.0445	0.6802	1.4701
MAPK3	57	0.0170	0.6573	1.5214
PPARG	57	0.0358	0.6573	1.5214
MMP9	57	0.0284	0.6573	1.5214
JUN	56	0.0178	0.6536	1.5299
PTGS2	54	0.0333	0.6464	1.5470
ESR1	54	0.0385	0.6464	1.5470
CCL2	52	0.0100	0.6393	1.5641
PPARA	46	0.0168	0.6158	1.6239
NOS3	45	0.0129	0.6158	1.6239
IL2	45	0.0172	0.6031	1.6581
CAT	44	0.0162	0.6126	1.6325

CCND1	43	0.0072	0.6094	1.6410
VCAM1	43	0.0049	0.6000	1.6667
ICAM1	42	0.0035	0.6000	1.6667
FOS	42	0.0221	0.6062	1.6496
RELA	41	0.0057	0.5969	1.6752
MAPK1	39	0.0092	0.5969	1.6752
MMP2	38	0.0053	0.5939	1.6838
MAPK14	38	0.0030	0.5909	1.6923
JAK2	35	0.0028	0.5821	1.7179
HMOX1	34	0.0020	0.5764	1.7350
NOS2	34	0.0020	0.5680	1.7607
IGF1R	33	0.0078	0.5764	1.7350
NFKB1	32	0.0024	0.5652	1.7692
CYP3A4	31	0.0215	0.5652	1.7692
APP	31	0.0096	0.5680	1.7607
GPT	31	0.0085	0.5652	1.7692
AR	29	0.0062	0.5652	1.7692
F2	29	0.0081	0.5571	1.7949
IFNB1	29	0.0015	0.5571	1.7949
CYP19A1	29	0.0137	0.5598	1.7863
SOD1	28	0.0077	0.5467	1.8291
SELE	27	0.0008	0.5442	1.8376

PARP1	27	0.0058	0.5467	1.8291
AGTR1	26	0.0087	0.5519	1.8120
NOX4	26	0.0008	0.5467	1.8291
ESR2	25	0.0051	0.5545	1.8034
ARG1	24	0.0009	0.5294	1.8889
ABCB1	23	0.0064	0.5367	1.8632
KIT	23	0.0007	0.5392	1.8547
TLR9	23	0.0010	0.5132	1.9487
IGFBP3	23	0.0035	0.5392	1.8547
DPP4	22	0.0058	0.5442	1.8376
PDGFRB	21	0.0003	0.5294	1.8889
GSR	20	0.0010	0.5223	1.9145
TERT	20	0.0013	0.5223	1.9145
ABCG2	19	0.0027	0.5318	1.8803
NR1I2	19	0.0029	0.5318	1.8803
ADRB2	19	0.0115	0.5318	1.8803
SIRT2	19	0.0013	0.5177	1.9316
CSF1R	19	0.0003	0.5223	1.9145
PRKACA	19	0.0035	0.5132	1.9487
TGM2	17	0.0006	0.5109	1.9573
LDLR	17	0.0003	0.5132	1.9487
PON1	16	0.0010	0.5109	1.9573

CFTR	16	0.0050	0.5109	1.9573
GHR	16	0.0017	0.5087	1.9658
CTSK	16	0.0021	0.5000	2.0000
RXRA	16	0.0014	0.5087	1.9658
MIF	15	0.0000	0.5132	1.9487
CXCR3	15	0.0001	0.4835	2.0684
ELANE	15	0.0004	0.4875	2.0513
HMGCR	14	0.0013	0.5087	1.9658
CYP2B6	14	0.0043	0.4606	2.1709
CYP1B1	14	0.0018	0.5087	1.9658
PDGFRA	14	0.0001	0.5065	1.9744
HSD11B1	14	0.0066	0.5021	1.9915
FLT3	13	0.0001	0.5000	2.0000
IGFBP1	13	0.0008	0.4979	2.0085
CALM1	13	0.0038	0.5000	2.0000
RAD51	11	0.0010	0.4815	2.0769
BCL2	11	0.0006	0.4835	2.0684
ALOX15	11	0.0030	0.4937	2.0256
HSD17B1	11	0.0013	0.4466	2.2393
SLC6A4	10	0.0031	0.4855	2.0598
ABCC1	10	0.0003	0.4895	2.0427
P4HB	10	0.0002	0.4937	2.0256

ESRRA	10	0.0001	0.5021	1.9915
MMP12	10	0.0001	0.4680	2.1368
IGFBP2	9	0.0001	0.4916	2.0342
MALT1	9	0.0000	0.4625	2.1624
CYP11B1	8	0.0006	0.4091	2.4444
CYP11B2	8	0.0007	0.4224	2.3675
IGFBP5	8	0.0004	0.4756	2.1026
TTR	8	0.0001	0.4855	2.0598
SRD5A1	8	0.0006	0.4194	2.3846
HTR2C	7	0.0005	0.4333	2.3077
CRHR1	7	0.0009	0.4398	2.2735
P2RX7	7	0.0016	0.4625	2.1624
FEN1	7	0.0005	0.4661	2.1453
HTR2A	6	0.0003	0.4149	2.4103
BAD	6	0.0001	0.4588	2.1795
F10	6	0.0001	0.4606	2.1709
IGFBP4	6	0.0000	0.4570	2.1880
PDE4D	5	0.0001	0.4606	2.1709
HPGD	5	0.0001	0.4349	2.2991
KCNJ11	5	0.0003	0.4535	2.2051
GBA	4	0.0171	0.4382	2.2821
ALDH2	4	0.0002	0.4077	2.4530

CA2	4	0.0001	0.4255	2.3504
ERCC5	4	0.0000	0.4194	2.3846
FABP3	4	0.0000	0.4500	2.2222
IGFBP6	4	0.0000	0.4382	2.2821
MGLL	4	0.0000	0.4535	2.2051
ALOX12	3	0.0000	0.4048	2.4701
KCNN3	3	0.0001	0.3926	2.5470
MTNR1B	3	0.0000	0.4415	2.2650
PREP	2	0.0000	0.4021	2.4872
GLB1	1	0.0000	0.3055	3.2735
PTPRS	1	0.0000	0.4063	2.4615

Table S4. The cluster network parameters.

Network Parameters	Value
Number of nodes	39
Number of edges	595
Clustering coefficient	0.868
Network diameters	2
Network density	0.803
Network radius	1
Network heterogeneity	0.192
Network centralization	0.208
Shortest paths	1482 (100%)
Avg. number of neighbors	30.513
Characteristic path length	1.197

Table S5. Data on cluster network.

Name	Degree	Betweenness Centrality	Closeness Centrality	Average Shortest Path Length
AKT1	80	0.0668	0.7548	1.3248
IL6	80	0.0592	0.7548	1.3248
INS	79	0.0919	0.7500	1.3333
TNF	76	0.0473	0.7358	1.3590
TP53	68	0.0650	0.7048	1.4188
VEGFA	67	0.0239	0.6964	1.4359
IL1B	65	0.0259	0.6882	1.4530
SRC	63	0.0445	0.6802	1.4701
MAPK3	57	0.0170	0.6573	1.5214
MMP9	57	0.0284	0.6573	1.5214
PPARG	57	0.0358	0.6573	1.5214
JUN	56	0.0178	0.6536	1.5299
PTGS2	54	0.0333	0.6464	1.5470
ESR1	54	0.0385	0.6464	1.5470
CCL2	52	0.0100	0.6393	1.5641
PPARA	46	0.0168	0.6158	1.6239
IL2	45	0.0172	0.6031	1.6581
NOS3	45	0.0129	0.6158	1.6239
CAT	44	0.0162	0.6126	1.6325

VCAM1	43	0.0049	0.6000	1.6667
CCND1	43	0.0072	0.6094	1.6410
ICAM1	42	0.0035	0.6000	1.6667
FOS	42	0.0221	0.6062	1.6496
RELA	41	0.0057	0.5969	1.6752
MAPK1	39	0.0092	0.5969	1.6752
MAPK14	38	0.0030	0.5909	1.6923
MMP2	38	0.0053	0.5939	1.6838
JAK2	35	0.0028	0.5821	1.7179
HMOX1	34	0.0020	0.5764	1.7350
NOS2	34	0.0020	0.5680	1.7607
IGF1R	33	0.0078	0.5764	1.7350
NFKB1	32	0.0024	0.5652	1.7692
APP	31	0.0096	0.5680	1.7607
GPT	31	0.0085	0.5652	1.7692
IFNB1	29	0.0015	0.5571	1.7949
SELE	27	0.0008	0.5442	1.8376
NOX4	26	0.0008	0.5467	1.8291
AGTR1	26	0.0087	0.5519	1.8120
ESR2	25	0.0051	0.5545	1.8034

Table S6. Annotation of biological processes.

Term ID	Description	*P* value	Gene count	Gene ratio (%)
GO:0071417	cellular response to organonitrogen compound	1.15E-40	69	57.98
GO:0010035	response to inorganic substance	4.79E-30	41	34.45
GO:0071407	cellular response to organic cyclic compound	5.50E-29	47	39.50
GO:0009991	response to extracellular stimulus	3.09E-27	45	37.82
GO:0043408	regulation of MAPK cascade	3.39E-27	48	40.34
GO:0034097	response to cytokine	3.39E-25	44	36.97
GO:0048545	response to steroid hormone	2.82E-24	27	22.69
GO:0031347	regulation of defense response	2.46E-23	40	33.61
GO:0010817	regulation of hormone levels	5.62E-23	40	33.61
GO:0045834	positive regulation of lipid metabolic process	1.17E-20	35	29.41
GO:0030335	positive regulation of cell migration	2.09E-20	32	26.89
GO:0006954	inflammatory response	2.19E-20	27	22.69
GO:0048732	gland development	2.57E-20	39	32.77
GO:0030155	regulation of cell adhesion	3.39E-19	47	39.50
GO:0007568	aging	6.46E-19	21	17.65
GO:0003013	circulatory system process	2.75E-18	28	23.53
GO:0008202	steroid metabolic process	4.90E-18	31	26.05
GO:0044057	regulation of system process	6.61E-18	32	26.89
GO:0070482	response to oxygen levels	1.07E-17	51	42.86

GO:0032355	response to estradiol	1.07E-17	19	15.97

Table S7. Annotation of molecular function.

Term ID	Description	*P* value	Gene count	Gene ratio (%)
GO:0020037	heme binding	1.86E-12	26	21.85
GO:0042803	protein homodimerization activity	3.09E-12	23	19.33
GO:0031994	insulin-like growth factor I binding	2.00E-11	14	11.76
GO:0004879	nuclear receptor activity	1.45E-08	20	16.81
GO:0050661	NADP binding	1.55E-08	9	7.56
GO:0140297	DNA-binding transcription factor binding	2.19E-08	20	16.81
GO:0002020	protease binding	5.50E-08	10	8.40
GO:0070851	growth factor receptor binding	6.03E-08	10	8.40
GO:0019904	protein domain specific binding	4.37E-07	25	21.01
GO:0019902	phosphatase binding	1.15E-06	10	8.40
GO:0004672	protein kinase activity	1.41E-06	15	12.61
GO:0004252	serine-type endopeptidase activity	5.75E-06	11	9.24
GO:0030545	signaling receptor regulator activity	5.75E-06	16	13.45
GO:0016922	nuclear receptor binding	0.00014	7	5.88
GO:0070412	R-SMAD binding	0.00022	4	3.36
GO:0047485	protein N-terminus binding	0.00037	14	11.76
GO:0033218	amide binding	0.00039	12	10.08
GO:0051378	serotonin binding	0.00112	4	3.36
GO:0042169	SH2 domain binding	0.00170	7	5.88

GO:0008559	ABC-type xenobiotic transporter activity	0.00170	8	6.72

Table S8. Annotation of cellular component.

Term ID	Description	*P* value	Gene count	Gene ratio (%)
GO:0045121	membrane raft	1.48E-11	22	18.49
GO:0098552	side of membrane	7.08E-10	20	16.81
GO:0005788	endoplasmic reticulum lumen	6.92E-09	14	11.76
GO:0045177	apical part of cell	3.31E-08	15	12.61
GO:0043235	receptor complex	8.51E-08	16	13.45
GO:0000323	lytic vacuole	7.24E-07	24	20.17
GO:0043025	neuronal cell body	4.79E-05	12	10.08
GO:0016942	insulin-like growth factor binding protein complex	5.75E-05	3	2.52
GO:0043209	myelin sheath	8.51E-05	5	4.20
GO:0048471	perinuclear region of cytoplasm	8.51E-05	14	11.76
GO:0005667	transcription regulator complex	0.00027	11	9.24
GO:0031904	endosome lumen	7.76E-04	6	5.04
GO:0036019	endolysosome	1.17E-02	5	4.20
GO:0030139	endocytic vesicle	0.02340	11	9.24
GO:0000781	chromosome, telomeric region	0.02884	6	5.04
GO:0043202	lysosomal lumen	0.03236	4	3.36
GO:0098794	postsynapse	0.03631	9	7.56
GO:0005911	cell-cell junction	0.03981	12	10.08

Table S9. Data on the target–pathway network in KEGG enrichment.

name	Degree	Betweenness Centrality	Closeness Centrality	Average Shortest Path Length
hsa05417	63	0.3826	0.5787	1.7281
hsa05200	59	0.3067	0.5561	1.7982
hsa05207	27	0.0656	0.4207	2.3772
hsa05206	19	0.0255	0.3972	2.5175
hsa04750	18	0.0321	0.3838	2.6053
hsa04668	18	0.0180	0.3891	2.5702
hsa04020	17	0.0338	0.3689	2.7105
hsa05415	14	0.0256	0.3666	2.7281
hsa04380	14	0.0079	0.3596	2.7807
hsa04211	13	0.0216	0.3689	2.7105
hsa04064	13	0.0193	0.3762	2.6579
hsa05202	13	0.0256	0.3619	2.7632
hsa04060	12	0.0094	0.3551	2.8158
hsa05144	12	0.0070	0.3465	2.8860
hsa04726	11	0.0332	0.3619	2.7632
AKT1	11	0.0209	0.4634	2.1579
hsa04923	10	0.0223	0.3738	2.6754
hsa04630	10	0.0046	0.3508	2.8509
hsa00140	10	0.0810	0.3486	2.8684

hsa04976	10	0.0189	0.3574	2.7982
RELA	10	0.0176	0.4597	2.1754
PRKACA	10	0.0371	0.4750	2.1053
NFKB1	10	0.0176	0.4597	2.1754
PTGS2	9	0.0639	0.4711	2.1228
hsa04913	8	0.0275	0.3642	2.7456
IL1B	8	0.0107	0.4419	2.2632
MAPK3	8	0.0131	0.4488	2.2281
MAPK1	8	0.0131	0.4488	2.2281
TNF	7	0.0072	0.4318	2.3158
INS	7	0.0175	0.4488	2.2281
IFNB1	7	0.0073	0.4318	2.3158
MAPK14	7	0.0091	0.4419	2.2632
JUN	7	0.0074	0.4385	2.2807
IL6	7	0.0095	0.4318	2.3158
FOS	7	0.0074	0.4385	2.2807
ICAM1	6	0.0064	0.4318	2.3158
VEGFA	6	0.0078	0.4351	2.2982
TP53	6	0.0081	0.4385	2.2807
RXRA	6	0.0104	0.4419	2.2632
PPARG	6	0.0088	0.4385	2.2807
MMP9	6	0.0068	0.4318	2.3158

BCL2	6	0.0060	0.4351	2.2982
CCND1	6	0.0062	0.4351	2.2982
CYP1B1	5	0.0242	0.4191	2.3860
VCAM1	5	0.0033	0.3826	2.6140
ADRB2	5	0.0049	0.3931	2.5439
PPARA	5	0.0054	0.4351	2.2982
PDGFRA	5	0.0050	0.4254	2.3509
JAK2	5	0.0049	0.4318	2.3158
CSF1R	5	0.0051	0.4254	2.3509
CALM1	5	0.0057	0.4351	2.2982
AGTR1	5	0.0067	0.4351	2.2982
CFTR	4	0.0020	0.3353	2.9825
CCL2	4	0.0019	0.3826	2.6140
CYP2B6	4	0.0211	0.4191	2.3860
HTR2C	4	0.0045	0.3904	2.5614
HTR2A	4	0.0045	0.3904	2.5614
LDLR	4	0.0091	0.4385	2.2807
NOS3	4	0.0047	0.4254	2.3509
GHR	4	0.0027	0.3750	2.6667
SRC	4	0.0029	0.4286	2.3333
PDGFRB	4	0.0022	0.3750	2.6667
NOS2	4	0.0034	0.4222	2.3684

IL2	4	0.0032	0.4191	2.3860
IGF1R	4	0.0059	0.3851	2.5965
CYP3A4	3	0.0080	0.3220	3.1053
ABCC1	3	0.0025	0.3878	2.5789
ALOX12	3	0.0029	0.3904	2.5614
PARP1	3	0.0016	0.3775	2.6491
SOD1	3	0.0017	0.3904	2.5614
CTSK	3	0.0009	0.3725	2.6842
SELE	3	0.0008	0.3725	2.6842
CRHR1	3	0.0015	0.3775	2.6491
ABCB1	3	0.0023	0.3800	2.6316
CAT	3	0.0022	0.4222	2.3684
IGFBP3	3	0.0025	0.4191	2.3860
MMP2	3	0.0022	0.4191	2.3860
HMOX1	3	0.0012	0.4161	2.4035
FLT3	3	0.0017	0.3725	2.6842
F2	3	0.0025	0.4191	2.3860
ESR2	3	0.0015	0.4222	2.3684
ESR1	3	0.0015	0.4222	2.3684
BAD	3	0.0015	0.4222	2.3684
CA2	2	0.0004	0.2969	3.3684
HMGCR	2	0.0003	0.2879	3.4737

HSD17B1	2	0.0007	0.2767	3.6140
CYP19A1	2	0.0007	0.2767	3.6140
ALOX15	2	0.0005	0.2923	3.4211
ELANE	2	0.0010	0.3775	2.6491
CXCR3	2	0.0007	0.3775	2.6491
APP	2	0.0017	0.3800	2.6316
P2RX7	2	0.0012	0.3904	2.5614
ABCG2	2	0.0018	0.3826	2.6140
MALT1	2	0.0006	0.3725	2.6842
TLR9	2	0.0005	0.3701	2.7018
MTNR1B	2	0.0006	0.3654	2.7368
PDE4D	2	0.0003	0.3701	2.7018
TERT	2	0.0007	0.4130	2.4211
KIT	2	0.0005	0.3654	2.7368
AR	2	0.0003	0.3701	2.7018
MGLL	1	0.0000	0.2727	3.6667
GPR35	1	0.0000	0.2701	3.7018
SRD5A1	1	0.0000	0.2591	3.8596
HSD11B1	1	0.0000	0.2591	3.8596
CYP11B2	1	0.0000	0.2591	3.8596
CYP11B1	1	0.0000	0.2591	3.8596
SLC6A4	1	0.0000	0.2664	3.7544

GSR	1	0.0000	0.2689	3.7193
HPGD	1	0.0000	0.2664	3.7544
ARG1	1	0.0000	0.3677	2.7193
ALDH2	1	0.0000	0.3677	2.7193
NOX4	1	0.0000	0.3677	2.7193
KCNN3	1	0.0000	0.3585	2.7895
KCNJ11	1	0.0000	0.3585	2.7895
RAD51	1	0.0000	0.3585	2.7895

Table S10. Zebrafish primer sets used for qPCR

Gene	Primer sequence（5' to 3'）	
	**Forward**	**Reverse**
CA2	AAGTGTGTTGCTGCCCAGTTCTC	AGACGATCCAGGTGACGCTCTC
CTSK	AGGGTAACGAGAGGGCACTGAC	GCGTGGTTGACATCTTCCTTATTGC
TRACP	TTGGCATGGATTGAGCAGAGACTTC	GTTGGACCGTGATGACCGATGG
MMP9	ACAACCACTGCTTCCACCACAAC	TGCGTTCACCATTGCCTGAGATC
P65	GGAACTTCGCAGGTGCCTCAAG	TTTCGTGGTGTCGTTGCTCTTCTC
MAPK14	TTCAGTCCATCATCCATGCCAAGC	ATCCAGCAAGCCGATCACATTCTC
TNF-α	CAAGGAGAACTAGCAGCATGGTGAG	GCAGCGAGTGTCTTCCATATCCAG
JUN	GAGAGCCAGGAGCGGATTAAAGC	TGCGACTTCAGGGTCTTGACTTTG
AKT1	AGAAGTTGGTTCCTCCGTTCAAGC	GTGGTGTGATGGTGATCGTCTGTC
ACTB	AACGAACGACCAACCTAAACCTCTC	CTCCCTTTCCAGTTTCCGCATCC
